# The significance of plagioclase textures in mid-ocean ridge basalt (Gakkel Ridge, Arctic Ocean)

**DOI:** 10.1007/s00410-019-1587-1

**Published:** 2019-05-21

**Authors:** Emma N. Bennett, C. Johan Lissenberg, Katharine V. Cashman

**Affiliations:** 1 School of Earth and Ocean Sciences, Cardiff University, Park Place, Cardiff, CF10 3AT UK; 2 School of Earth Sciences, University of Bristol, Wills Memorial Building, Bristol, BS8 1RJ UK

**Keywords:** Plagioclase, Mid-ocean ridge, Gakkel Ridge, Textures, Crystal mush, Magma

## Abstract

Textures and compositions of minerals can be used to infer the physiochemical conditions present within magmatic systems. Given that plagioclase is an abundant phase in many magmatic systems, understanding the link between texture and process is vital. Here, we present a database of textural and compositional data for > 1800 plagioclase crystals in mid-ocean ridge basalt from the Gakkel Ridge (Arctic Ocean) to investigate the physiochemical conditions and processes that govern the formation of plagioclase textures and compositions. The Gakkel basalts have high modal crystal contents (up to 50%). The crystal cargo is complex, with both individual plagioclase and glomerocrysts showing large variations in crystal habit, zoning and resorption. The most common types of zoning are reverse and patchy; we attribute patchy zoning to infilling following either skeletal growth or resorption. Resorption is abundant, with multiple resorption events commonly present in a single crystal, and results from both magmatic recharge and decompression. Periods of strong undercooling, distinct to quench crystallisation, are indicated by matured skeletal crystals and thin normally zoned melt inclusion-rich bands following resorption. Individual samples often contain diverse textural and compositional plagioclase groups. Furthermore, most plagioclase is not in equilibrium with its host melt. Finally, the porous open structures of some glomerocrysts suggest that they represent pieces of entrained disaggregated mush. We interpret this to indicate that the crystal cargo is not generally phenocrystic in origin. Instead, plagioclase crystals that formed in different parts of a mush-dominated plumbing system were entrained into ascending melts. The textures of individual crystals are a function of their respective histories of (under)cooling, magma mixing and decompression. The morphologies of melt inclusion trapped in the plagioclase crystals are associated with specific host crystal textures, suggesting a link between plagioclase crystallisation processes and melt inclusion entrapment. The database of plagioclase presented herein may serve as a template for the interpretation of plagioclase textures in magmatic systems elsewhere.

**Electronic supplementary material:**

The online version of this article (10.1007/s00410-019-1587-1) contains supplementary material, which is available to authorized users.

## Introduction

Textures and compositions of minerals record the physiochemical conditions and processes occurring within magmatic systems (Vance 1965; Meyer and Shibata 1990; Ginibre et al. 2002; Pan and Batiza 2002; Pietranik et al. 2006; Ridley et al. 2006; Ginibre and Wörner 2007; Hellevang and Pedersen 2008; Viccaro et al. 2010; Cashman and Blundy 2013; Neave et al. 2014; Coote and Shane 2016; Bouvet de Maisonneuve et al. 2016). Plagioclase, in particular, is useful for investigating magmatic processes because, in addition to being an abundant phase in lavas from a range of geotectonic settings (e.g. continental arcs (Ginibre et al. 2002; Cashman and Blundy 2013); ocean islands (Cullen et al. 1989); mid-ocean ridges (Meyer and Shibata 1990; Nielsen et al. 1994; Lange et al. 2013; Drignon et al. 2018); layered intrusions (Maaløe 1976), the slow inter-diffusion of CaAl–NaSi prevents equilibration of adjacent compositional zones (Morse 1984; Grove et al. 1984). This slow diffusion preserves textures over long timescales, providing petrologists with an observable record of the processes occurring within a magmatic system. Mid-ocean ridges are an ideal end-member magmatic system in which to study plagioclase textures because, unlike wet, compositionally variable volcanic arcs, mid-ocean ridge basalts (MORB) have relatively uniform compositions (i.e. tholeiitic basalts), have low water contents (Michael 1995; Danyushevsky 2001) and either experience variable (Soule et al. 2012) or no volatile degassing (Saal et al. 2002); pyroclastic deposits at 85°E on the Gakkel Ridge provide evidence that in some instances volatile degassing can occur (e.g. Sohn et al. 2008). The relative compositional simplicity of this system is advantageous, because fewer variables need to be considered when reconciling plagioclase textures with magmatic processes.

Through the use of plagioclase, mid-ocean ridges have been shown to be dynamic systems within which multiple processes operate. Other than the important role that fractional crystallisation plays in the chemistry of MORB, two other processes shown to be important at a range of spreading rates are magma mixing and mush disaggregation. Multiple lines of petrological evidence support the occurrence of magma mixing: (1) mixed chemical and textural plagioclase populations (Dungan et al. 1979; Meyer and Shibata 1990; Pan and Batiza 2003); (2) plagioclase morphologies indicative of disequilibrium (i.e. resorption and skeletal grwth) (Kuo and Kirkpatrick 1982); (3) the presence of plagioclase that is too anorthitic to be in equilibrium with its host melt (e.g. Dungan et al. 1979); and reverse zoning (e.g. Dungan et al. 1978; Hellevang and Pedersen 2008). Plagioclase diffusion studies also suggest that at some mid-ocean ridges, magma mixing and replenishment actively cause mush disaggregation (Costa et al. 2009; Moore et al. 2014). Mush zone disaggregation is attributed to the presence of anorthitic plagioclase xenocrysts (Ridley et al. 2006) and open-structured crystal networks (Pan and Batiza 2003) in MORB, as well as the formation of plagioclase ultraphyric basalts (PUBs) commonly sampled at ultraslow- to intermediate-spreading ridges (Lange et al. 2013). The importance of interactions between percolating melt and pre-existing crystal frameworks within mush zones is demonstrated by textures of individual plagioclase (Coumans et al. 2015) and plagioclase in cumulate xenoliths (Ridley et al. 2006).

Understanding plagioclase growth mechanisms and textures also has implications for the interpretation of melt inclusions, because disequilibrium processes that form melt inclusions may act to modify their compositions (Nakamura and Shimatika 1998; Danyushevsky et al. 2002; Michael et al. 2002). During skeletal growth and resorption, a chemical boundary layer may form at the melt–crystal interface enriched in plagioclase incompatible (e.g. Fe, Mg, Ti) (Bottinga et al. 1966) and compatible (e.g. Ca, Na) (Nakamura and Shimatika 1998; Danyushevsky et al. 2002) elements, respectively. In both cases, the composition of the final melt inclusion depends on element diffusivities being rapid enough to dissipate the boundary layer prior to melt inclusions becoming occluded (Danyushevsky et al. 2002). Understanding the processes that form melt inclusions and associated textures (e.g. Nakamura and Shimatika 1998; Faure and Schiano 2005) is therefore vital in order to use melt inclusion compositions to determine the compositional heterogeneity of melts within magmatic systems as is commonly done (e.g. Nielsen et al. 1994; Kamenetsky et al. 1998; Sours-Page et al. 1999; Maclennan 2008).

Whilst studies of mid-ocean ridge plagioclase exist (Dungan et al. 1978; Kuo and Kirkpatrick 1982; Meyer and Shibata 1990; Pan and Batiza 2003; Ridley et al. 2006; Hellevang and Pedersen 2008; Lange et al. 2013), there are currently no studies that rigorously investigate the significance of different plagioclase textures and/or place quantitative constraints on the relative importance of the processes that form the textures. Additionally, whilst plagioclase has been used to track magmatic processes at fast- and slow-spreading ridges such as the East Pacific Rise (Pan and Batiza 2003; Ridley et al. 2006; Zhang et al. 2008; Moore et al. 2014; Zeng et al. 2014) and Mid-Atlantic ridge (Rhodes et al. 1979; Flower 1980; Kuo and Kirkpatrick 1982; Meyer and Shibata 1990; Faure and Schiano 2004; Costa et al. 2009; Lange et al. 2013), plagioclase from ultraslow-spreading ridges, such as the Gakkel Ridge, has received comparatively little attention (Hellevang and Pedersen 2008; Zellmer et al. 2011, 2012). These ultraslow-spreading ridges are of interest because, not only are they a spreading ridge end member, but they are also volumetrically significant, with ridges exhibiting ultraslow-spreading ridge characteristics making up ~ 36% of the Global mid-ocean ridge  system (Dick et al. 2003).

**Fig. 1:**
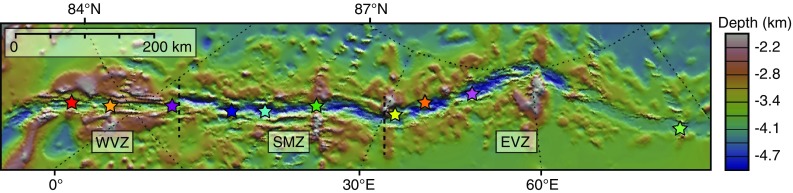
Bathymetric map of the Gakkel Ridge showing the locations of each tectono-magmatic segment defined by Michael et al. (2003). Sampling locations are shown by the coloured stars. Bathymetric data from Jakobsson et al. (2012) and Michael et al. (2003). Map made using GeoMapApp (http://www.geomapapp.org)

Here, using a large catalogue of back-scattered electron (BSE) images in combination with mineral major and trace element data, we present a systematic quantitative study of plagioclase from the ultraslow-spreading Gakkel Ridge (Fig. 1), with the aim of identifying what processes occur within the magma plumbing system and what their relative importance is. We will show that plagioclase textures are complex and can be linked to the occurrence of multiple processes within the magmatic plumbing system, including magma mixing, mush disaggregation, undercooling and decompression. In addition, the correlation between melt inclusion morphology and host crystal textures indicates that different morphologies result from different processes. We propose that within the Gakkel Ridge, the two dominant processes are magma mixing and mush disaggregation.

## Controls on plagioclase crystallisation

**Fig. 2:**
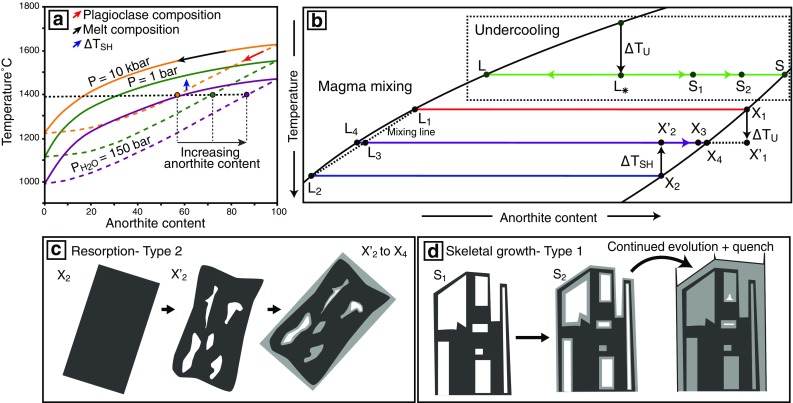
The controls on plagioclase composition and morphology. **a** Schematic phase diagram in the albite-anorthite system showing the effect of changing intensive variables (e.g. temperature, pressure and water content) on plagioclase composition. At the same temperature, plagioclase anorthite content increases with increasing and decreasing water content (purple) and pressure (green), respectively. **b** Schematic phase diagram in the albite–anorthite system showing the effect of (1) undercooling and (2) mixing between two liquids (*L*_1_ and *L*_2_ to form *L*_3_) on both plagioclase composition and morphology. During magma mixing, pre-existing plagioclase (*X*_2_) experience superheating (∆*T*_SH_) and resorption (*X*_2_–$X_{2}^{\prime }$). If plagioclase is not completely dissolved, the resorbed plagioclase becomes overgrown and infilled with more anorthitic plagioclase (i.e. reverse zoning) as the system moves towards equilibrium; Type 2 patchy zoning is formed ($X_{2}^{\prime }$–*X*_4_) (**c**). Newly formed plagioclase (*X*_3_) also grown from *L*_3_ and become reverse zoned (*X*_3_–*X*_4_). Magma mixing can also result in undercooling (∆*T*_U_) of pre-exiting plagioclase (*X*_1_–$X_{1}^{\prime }$). Type 1 patchy zoning (**d**) results from initial undercooling (∆*T*_U_ to produce L_*_) resulting in the formation of skeletal crystals (*S*_1_) whose composition lies between L_*_ and *S*. Both solid and liquid phases will evolve towards equilibrium (*L* and *S*, respectively). As the system evolves (*S*_1_–*S*), the skeletal framework becomes infilled through continued crystallisation; the plagioclase become reverse zoned and matured skeletal crystals exhibiting Type 1 patchy zoning are formed. Phase diagrams are re-drawn from Yoder (1968) (**a**), Lofgren (1974) (undercooling in **b**), and Kuo and Kirkpatrick (1982) (magma mixing in **b**)

The composition and morphology of plagioclase is controlled by changes in temperature, pressure, melt composition (including water content) and growth, dissolution and nucleation kinetics. The effect these have on plagioclase compositions and morphologies can be visualised using phase diagrams (Fig. 2a, b). At constant pressure and low water content, decreasing temperature drives crystallisation which can cause magmatic differentiation (black arrow in Fig. 2a). As differentiation proceeds, the corresponding equilibrium plagioclase composition becomes progressively more sodic (red arrow in Fig. 2a). Alternatively, if the ambient temperature of the system increases, with no corresponding change in bulk melt composition (e.g. from an adjacent or underlying melt intrusion), pre-existing plagioclase may become superheated (∆*T*_SH_) with respect to the solidus and become resorbed (blue arrow in Fig. 2a). Resorption within dry systems such as mid-ocean ridges can also occur during H_2_O-undersaturated decompression (Nelson and Montana 1992). At high pressures, equilibrium plagioclase is more sodic than at lower pressures (Yoder 1968; Panjasawatwong et al. 1995; Ustunisik et al. 2014) (Fig. 2a); hence, decompression from high to low pressures (yellow–green lines in Fig. 2a) causes resorption and crystallisation of more calcic plagioclase (i.e. reverse zoning) (Nelson and Montana 1992). In contrast, decompression under water-saturated conditions results in degassing and crystallisation of more sodic plagioclase (Blundy and Cashman 2005). Whilst the addition of water depresses the plagioclase loop causing the equilibrium plagioclase composition to become more anorthitic (green–purple lines in Fig. 2a) (e.g. Yoder 1968), the effect of water in mid-ocean ridge systems is small because MORB have uniformly low water contents (Michael 1995).

Magma mixing can change both the bulk composition and temperature of the magmatic system. Figure 2b shows how mixing between melts of composition *L*_1_ and *L*_2_ can cause periods of disequilibrium that can trigger undercooling (∆*T*_U_, *X*_1_–$X_{1}^{\prime }$) and/or superheating (i.e. resorption) (∆*T*_SH_, *X*_2_–$X_{2}^{\prime }$). Both plagioclase composition and morphology have been shown experimentally to be a function of the degree of undercooling (Lofgren 1972, 1974). Increasing the degree of undercooling has multiple effects: (1) a progressive change from tabular (at equilibrium) to skeletal, dendritic and eventually spherulitic plagioclase morphologies; and (2) crystallisation of plagioclase that is more sodic than at equilibrium. Crystallisation experiments of Lofgren (1974) have produced skeletal plagioclase crystals under conditions of strong undercooling that show reverse zoning. This reverse zoning is proposed to be the result of the crystal attempting to re-attain equilibrium growth conditions (Smith and Lofgren 1979) (*S*_1_–*S*) (Fig. 2b). Rapid growth can also cause disequilibrium partitioning of minor and trace elements to form a chemical boundary layer at the plagioclase–melt interface (Bottinga et al. 1966) enriched in plagioclase-incompatible elements (e.g. Mg, Fe, Ti). During continued crystal growth, these incompatible elements can become incorporated into the crystal at concentrations different from that expected during equilibrium crystallisation. Within mid-ocean ridges, three additional processes have been suggested to cause undercooling; (1) quench crystallisation upon eruption (Kirkpatrick and Jolla 1976); (2) intrusion of melts into a cool region of the plumbing system (Meyer and Shibata 1990; Hellevang and Pedersen 2008); and (3) nucleation delay which has been attributed to the formation of skeletal plagioclase found in oceanic gabbros drilled from the Cocos Plate (e.g. Koepke et al. 2011). Magma mixing can also cause partial dissolution resulting in the formation of dissolution–reprecipitation reaction textures (e.g. Nakamura and Shimatika 1998).

## Geological background and sample details

The Gakkel Ridge, located within the Arctic Ocean, is the ultraslow-spreading end member in the global mid-ocean ridge system (Fig. 1); the spreading rate decreases from 14.6 to 6.3 mm year^−1^ from west to east along the ridge (DeMets et al. 1990). Based on bathymetric data and recovered sample lithologies, the ridge can be split into three tectono-magmatic segments that exhibit alternating modes of magmatic and amagmatic crustal accretion (Michael et al. 2003; Dick et al. 2003): (1) a magmatically robust western volcanic zone (WVZ) characterised by inward dipping normal faults and axial volcanic ridges; (2) a central sparsely magmatic zone (SMZ) characterised by abundant peridotite exposed on the seafloor; and (3) an eastern volcanic zone (EVZ) characterised by widely spaced circular volcanic centres (Michael et al. 2003; Dick et al. 2003). Studies of basaltic glasses show that glasses from the WVZ have lower Mg# and Na_8.0_ compared to those from both the SMZ and EVZ suggesting that more extensive fractionation and higher degrees of partial melting occur here (Michael et al. 2003). In addition, Ba/TiO_2_ ratios are variable along the ridge and extend to the highest values within the SMZ at ~ 11°E, which suggests that the Gakkel Ridge mantle source is variably enriched (Michael et al. 2003). Similarly, the presence of a distinct isotopic boundary within the SMZ (Goldstein et al. 2008) and melt inclusion compositions (Wanless et al. 2014) support the presence of a heterogeneous mantle beneath the Gakkel Ridge. Samples used in this study come from ten volcanic systems from the full length of the ridge (Fig. 1).

## Methods

### Image acquisition, modal mineralogy and mineral and glass major element analysis

Plagioclase imaging and major element analysis were carried out using a Zeiss Sigma HD Field Emission Gun analytical scanning electron microscope (ASEM) outfitted with dual 150 mm^2^ Oxford X-MaxN silicon drift detector energy-dispersive spectrometers in the School of Earth and Ocean Science, Cardiff University (UK). Initial back-scattered electron (BSE) images of 1810 plagioclase crystals (individual, 1266; monomineralic glomerocryst, 167; polymineralic glomerocryst, 377) from 64 polished blocks were collected before quantitative microanalyses were acquired of a representative sub-sample of 670 plagioclase crystals. Quantitative analysis used a 1 nA beam current, 20.0 kV accelerating voltage, a fixed working distance of 8.9 mm and a 60 nm aperture. Plagioclase EDS spectra were acquired over 20–30 s live time using both detectors with an output count rate of 136 kcps. A defocused beam rastering over 5–10 μm^2^ areas minimised Na loss in plagioclase. Oxford Instruments Aztec Software was used to process and quantify raw data using the internal XPP matrix correction. A comprehensive suite of MAC and ASTIMEX primary standards were used to calibrate EDS analysis (Online Resource 2, Table S1). ASTIMEX plagioclase was used as secondary standard; standard analysis was accurate to within 0.8% An, with a precision of ± 0.34 An (accuracy and precision of standard measurements are reported in Online Resource 1). The plagioclase BSE images and compositional data were supplemented by 34 host glass analyses, as well as modal analysis of phenocrysts in 94 lavas using EDS element maps. Both the glass data and modal proportions are reported in Lissenberg et al. (2019).

### LA-ICPMS analysis

Plagioclase TiO_2_ were measured at the National Oceanography Centre and the University of Southampton (UK) using a New Wave UP193FX laser ablation system coupled to an HR-ICP-MS Thermo Fisher Scientific ELEMENT 2XR mass spectrometer. Beam spot sizes of 10 μm, 20 μm and 50 μm were used depending on the size of the feature being analysed. The laser fired with a frequency of 5 Hz at 80% energy over 35 s. Gas blanks were run at the start and end of each sample run, with standards (NIST 610, NIST 612, BCR-2G and BHVO-2G) analysed at the start of each sample run. Data were processed according to spot size; data were additionally gas blank corrected, with the CaO content determined through ASEM analysis serving as an internal standard. Targets were chosen following acquisition of BSE images and quantitative ASEM data. The accuracy and precision of standard measurements are reported in Online Resource 1.

### Plagioclase classification

The 1810 plagioclase BSE images were classified by their textural characteristics. The greyscale of these BSE images relates to the plagioclase anorthite (An) content (Ca/(Ca + Na) × 100); An content increases from dark grey to light grey. Individual plagioclase crystals > 1 and < 1 mm in size will be referred to throughout as macrocrysts and microcrysts, respectively: these terms have no genetic connotation. Monomineralic glomerocrysts are crystal aggregates containing > 2 similar-sized plagioclase crystals, whilst polymineralic glomerocrysts have > 2 phases of similar size. The full crystal database can be found in Online Resource 1. Classification schemes for individual plagioclase crystals and glomerocrysts can be found in Online Resource 2 (Table S2) and include, but are not restricted to, the parameters outlined in Table 1. Additional parameters used are as follows:

**Table 1:**
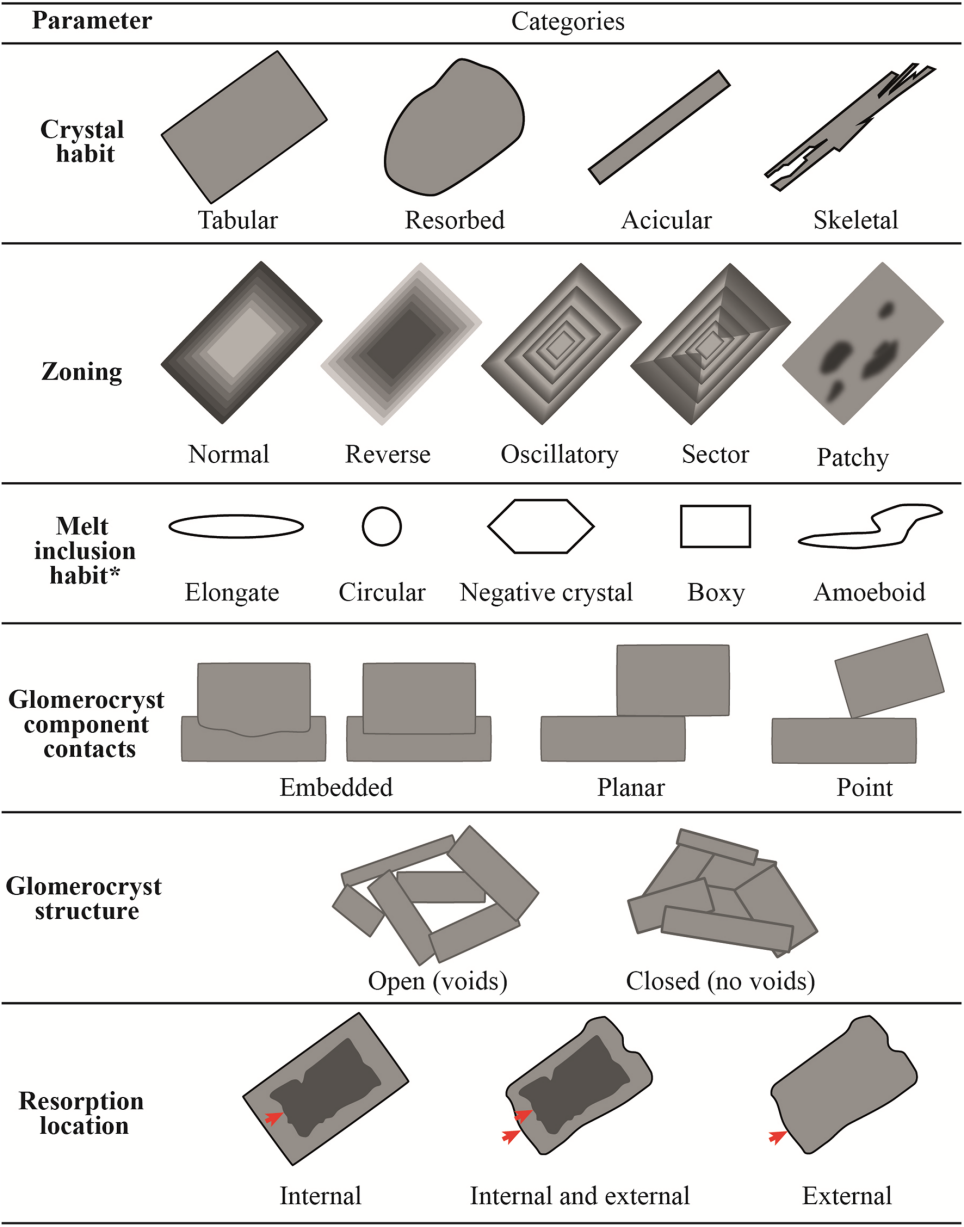
Plagioclase classification

*The term melt inclusion here is used to describe any void, whether glassy or crystalline, present within plagioclase

- Size measured as the maximum (apparent) crystal dimension.
- Aspect ratio.
- Zoning complexity. Crystals may exhibit more than one type of zoning: the zoning complexity is defined as the sum of the different zoning types present in a given crystal. For example, a crystal with both normal and reverse zoning would have a zoning complexity of 2, whilst one with normal, reverse and oscillatory zoning would have a value of 3. Patchy zoning is classified on an intensity scale of 1–5, a crystal with normal zoning and patchy zoning of intensity 5 would have a zoning complexity of 6. Patchy zoning of intensity 1 has low patch density, whilst patchy zoning of 5 has high patch density (Fig. S1). Zoning complexity is used as a proxy for the complexity of the magmatic conditions which an individual plagioclase crystal has experienced.

## Results

Each plagioclase and glomerocryst has been classified using the parameters outlined in “Plagioclase classification”; the full database can be found in Online Resource 1. In the following, we highlight results that are both the most significant and provide the most insight into the processes occurring in the magmatic system. Summary tables of individual parameters, along with relevant supplementary figures, can be found in Online Resource 2.

### Crystal cargo inventory

**Fig. 3:**
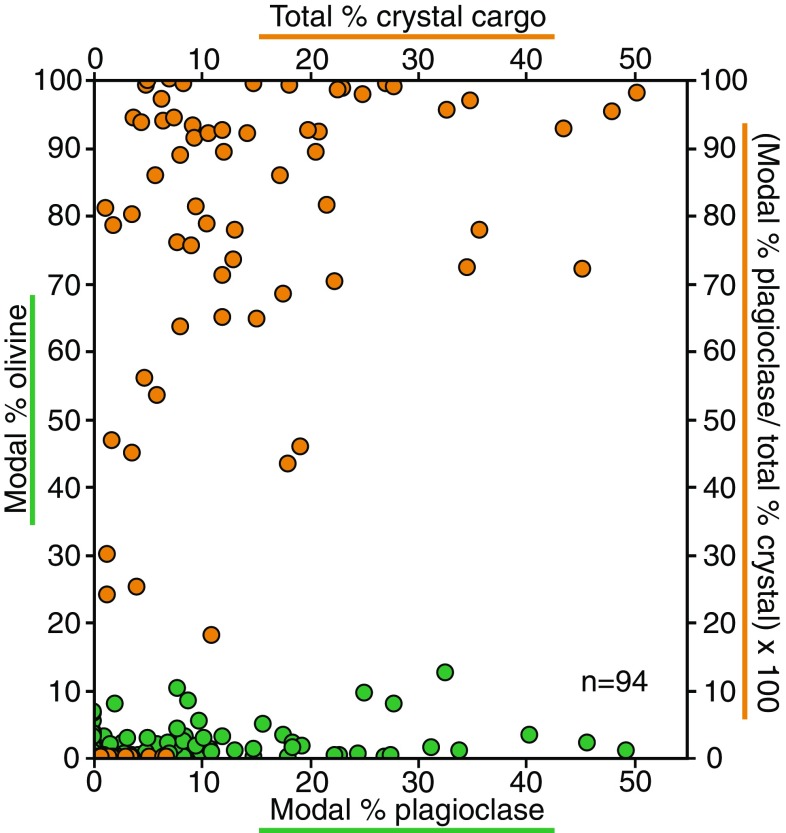
Relationships between total crystal cargo % and proportion of plagioclase [(modal % plagioclase/total % crystals) × 100)] (orange) and the relationship between the modal % of olivine and plagioclase (green). Samples analysed contain more plagioclase than olivine, and as the total crystal content increases, plagioclase content increases

The basaltic lavas sampled from the Gakkel Ridge range from aphyric to plagioclase phyric, having a total crystal content (e.g. plagioclase + olivine + clinopyroxene macrocrysts and microcrysts) between 0.4 and 50% (Fig. 3) with an average of 11% and a median of 6%. Modal plagioclase content ranges from 0 to 49% (average 9%). Figure 3 shows that plagioclase is the dominant mineral phase, and its proportion of the total crystal population [(modal % plagioclase/total % crystals) × 100)] increases with increased phenocryst content; modal olivine contents are 0–13% (average 2%). Spinel and clinopyroxene are the least common mineral phases, only present in a small number of samples (9 and 13 basalts, respectively).

### Textural observations

#### Crystal habits

**Fig. 4:**
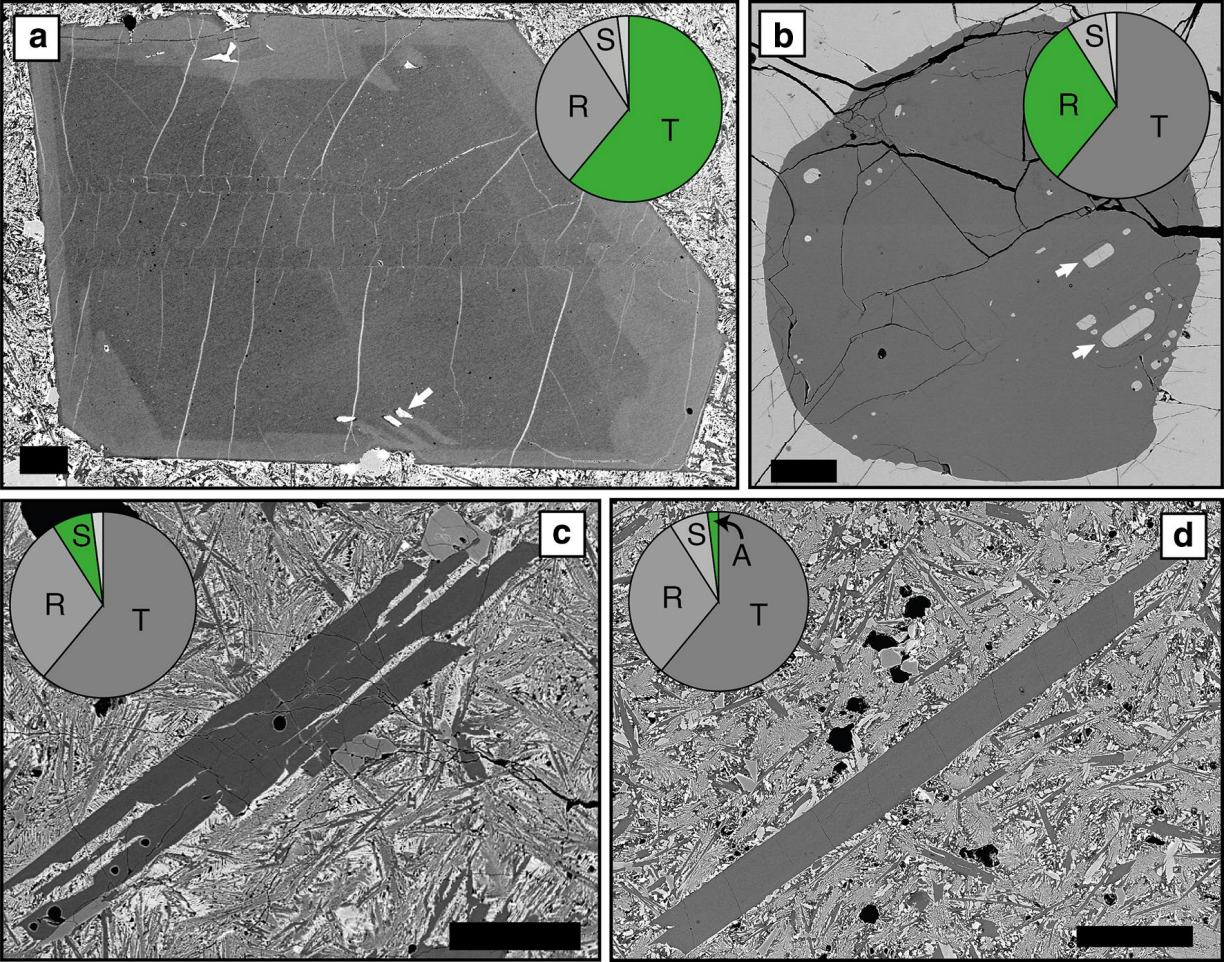
Plagioclase crystal habits. **a** Tabular plagioclase showing internal resorption and reverse zoning. **b** Resorbed plagioclase with no zoning. **c** Skeletal plagioclase. **d** Acicular plagioclase. Pie charts illustrate the proportions of each crystal habit present in the entire individual plagioclase database. White arrows in **a** and **b** indicate melt inclusions. Pie chart abbreviations are as follows: *T* tabular, *R* resorbed, *S* skeletal, *A* acicular. All scale bars are 0.25 mm

**Fig. 5:**
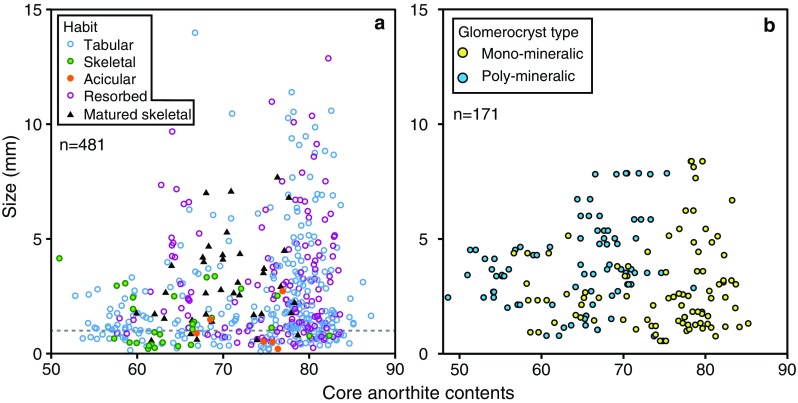
Relationship between habit, core anorthite contents and the size of both individual plagioclase (**a**) and glomerocrysts (**b**). **a** Tabular and resorbed plagioclase have the greatest range in sizes and cluster at higher anorthite content compared to skeletal and acicular crystals which are both smaller and generally restricted to lower anorthite content. **b** Poly- and monomineralic glomerocrysts have a similar size range; however, poly- and monomineralic glomerocrysts extend to lower and higher anorthite contents, respectively. Note: (1) where more than one measurement was taken of an individual plagioclase core, the values in **a** are an average; (2) despite the anorthite content of multiple components in a glomerocrysts being measured, the size used is the overall size of the glomerocryst, not the size of the individual plagioclase. The grey line in **a** represents the change from microcryst (< 1 mm) to macrocrysts (> 1 mm)

**Fig. 6:**
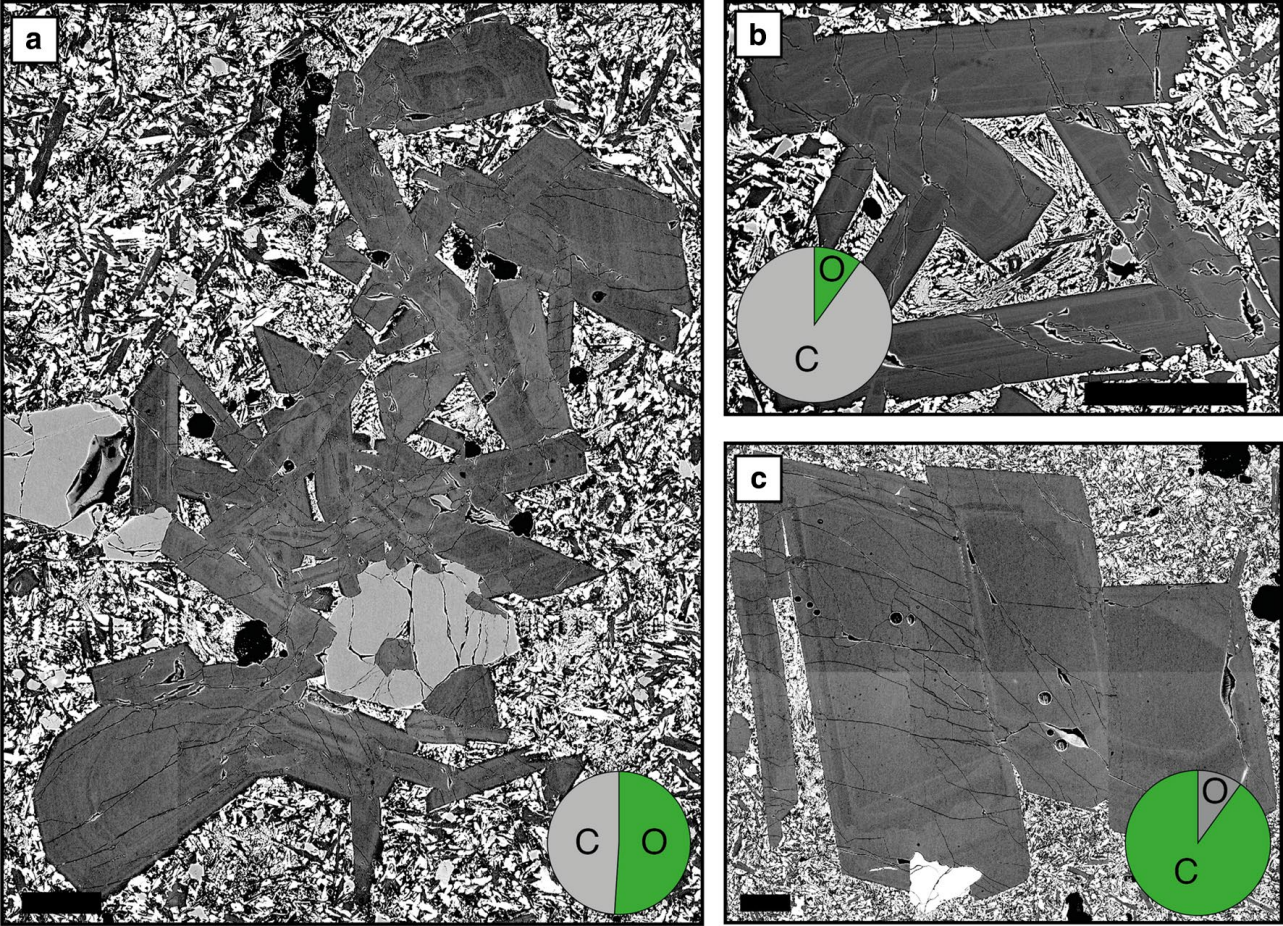
Glomerocryst configuration and contacts. **a** Polymineralic glomerocryst showing an open structure with plagioclase at high angles to one another. Monomineralic glomerocrysts show both open (**b**) and closed structures (**c**) characterised by high and low (planar) angle component contacts, respectively. Pie charts illustrate the proportions of each type of glomerocryst configuration in all mono- and polymineralic glomerocrysts in the database. Pie chart abbreviations are as follows: *O* open, *C* closed. All scale bars are 0.25 mm

Plagioclase shows a range of crystal morphologies (Fig. 4). The most abundant and largest crystals are those with tabular habits (Figs. 4a, 5a). Resorbed crystals have a similar size range (Fig. 5a) and comprise ~ 1/3 of all individual plagioclase (Fig. 4b). In comparison, individual skeletal and acicular crystals are both rarer (Fig. 4c, d) and smaller (Fig. 5a). Similar to individual plagioclase crystals, tabular and resorbed plagioclase are the most common components in monomineralic glomerocrysts (e.g. Fig. 6b, c; Table S3). Acicular and skeletal components are less common and are each only present in 5% of monomineralic glomerocrysts. In contrast, the most common crystal habits in polymineralic glomerocrysts are tabular and skeletal.

#### Zoning

**Fig. 7:**
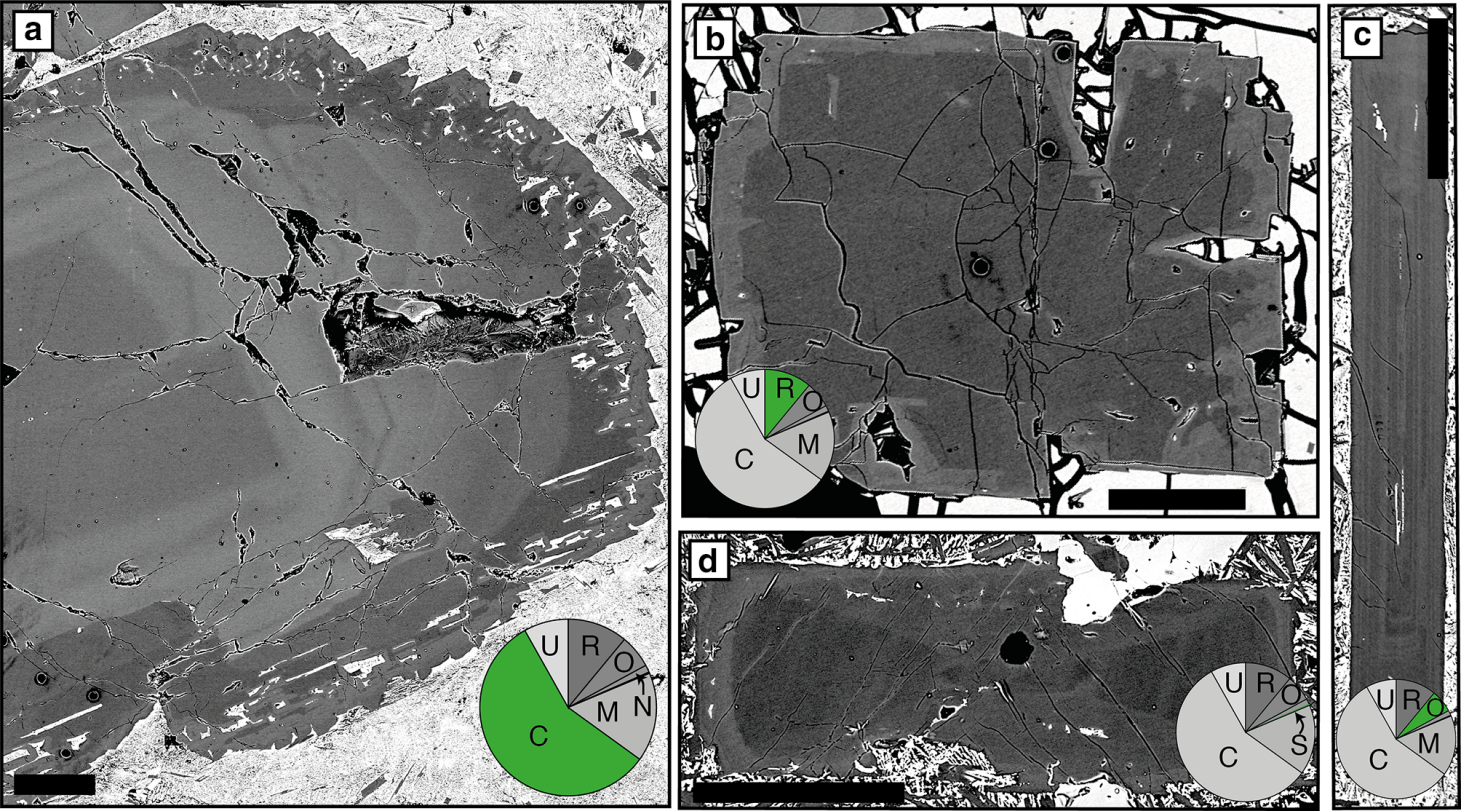
Variation in plagioclase zoning. **a** An example of complex zoning combinations in plagioclase including reverse, normal, oscillatory and patchy zoning. **b** Reverse zoning. **c** Oscillatory zoning. **d** A plagioclase showing sector and reverse zoning. Pie charts illustrate the proportions of each type of zoning; this is restricted to plagioclase in the entire database that exhibits only one type of zoning. Complex zoning includes plagioclase that has > 1 type of zoning present. Pie chart abbreviations are as follows: *C* complex, *U* unzoned, *R* reverse, *O* oscillatory, *N* normal, *S* sector, *P* patchy. All scale bars are 0.25 mm

**Fig. 8:**
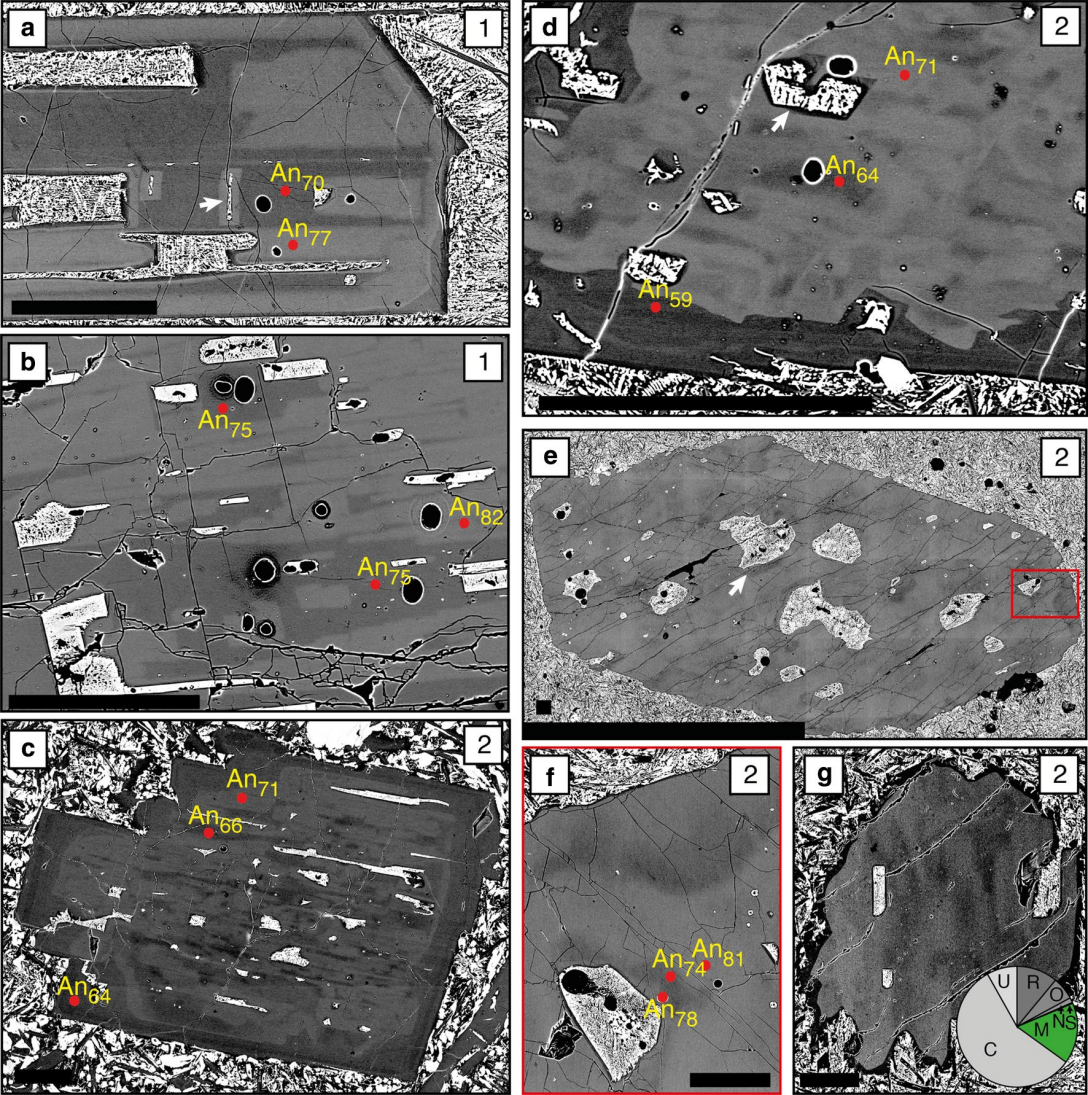
Type 1 (**a**, **b**) and Type 2 (**c**–**g**) patchy zoning in plagioclase; the zoning type is indicated in the top right of each panel. **a** Geometric Type 1 zoning with a skeletal geometric low anorthite core containing patches of higher anorthite similar in composition to the surrounding mantle. **b** The low anorthite skeletal core contains patches of higher anorthite, and higher anorthite regions also contain low anorthite patches similar in composition to the skeletal core. **c** Plagioclase exhibiting Type 2 patchy zoning characterised by low anorthite amoeboid patches that show a degree of crystallographic alignment along cleavage planes. These patches have similar anorthite contents to the outer region of the crystal. **d** Resorbed high anorthite core exhibiting Type 2 patchy zoning and outer oscillatory zoning. **e** Plagioclase exhibiting Type 2 patchy zoning. Here individual patches are present as well as voids that show zoning around their margins (**f**) similar to zoning on the exterior of the crystal. **g** Plagioclase showing a mottled core. White arrows indicate melt inclusions. The pie chart in **g** shows the proportions of each zoning type present in the entire individual plagioclase database. Pie chart abbreviations are as follows: *C* complex, *U* unzoned, *R* reverse, *O* oscillatory, *N* normal, *S* sector, *P* patchy. All scale bars are 0.25 mm

Complex zoning in individual crystals is the norm (e.g. Fig. 7a): only 19% contain one type of zoning (e.g. Fig. 7b, c) and 8% are unzoned (Table S4). Where plagioclase exhibits only one type of zoning, reverse (Fig. 7b) and patchy (Fig. 8e) zoning are the most common types (Table S5); in plagioclase exhibiting multiple types of zoning, reverse zoning is the most common (Table S6). The remainder of the crystals have complex zoning combinations (e.g. Figs. 7a, 8c, d). Sector zoning such as that shown in Fig. 7d is the least common zoning type in individual crystals. An important result is that tabular and resorbed crystals have higher zoning complexity (0–10; median 2) than skeletal and acicular crystals (0–5; median 1 and 0, respectively). There is no clear relationship between crystal size and zoning complexity (Fig. S2).

As with individual crystals, glomerocrysts often exhibit complex zoning; only 3–4% of glomerocrysts (poly- and monomineralic, respectively) have unzoned components (Tables S4, S5). Oscillatory zoning (Fig. 6a) is the most common individual zoning type in both mono- and polymineralic glomerocrysts. Zoning in monomineralic glomerocrysts is more complex than that in polymineralic glomerocrysts (e.g. Fig. 6c) (Tables S4, S5). Sector zoning, as with individual plagioclase, is the least common type of zoning in glomerocrysts.

Two distinct end-member types of patchy zoning are identified in both individual plagioclase and glomerocryst components: geometric/boxy (Type 1) (Fig. 8a, b) and amoeboid (Type 2) (Fig. 8c–g). Type 1 zoning is common in individual plagioclase that has low An skeletal cores (e.g. Fig. 8a, b); here these are termed matured skeletal crystals. Type 2 patchy zoning is found in plagioclase that shows evidence of resorption (e.g. Figs. 7a, 8c–g). Patches in both types of zoning are often closely associated with melt inclusions; boxy and amoeboid patches are associated with boxy (Fig. 8a, b) and amoeboid melt inclusions (Figs. 7a, 8c–f), respectively. Patches can be either higher or lower An than the surrounding plagioclase (e.g. Fig. 8b) and, whilst more often randomly distributed throughout the crystal (e.g. Fig. 8e), patches can show a degree of crystallographic alignment (Fig. 8c).

#### Resorption

**Fig. 9:**
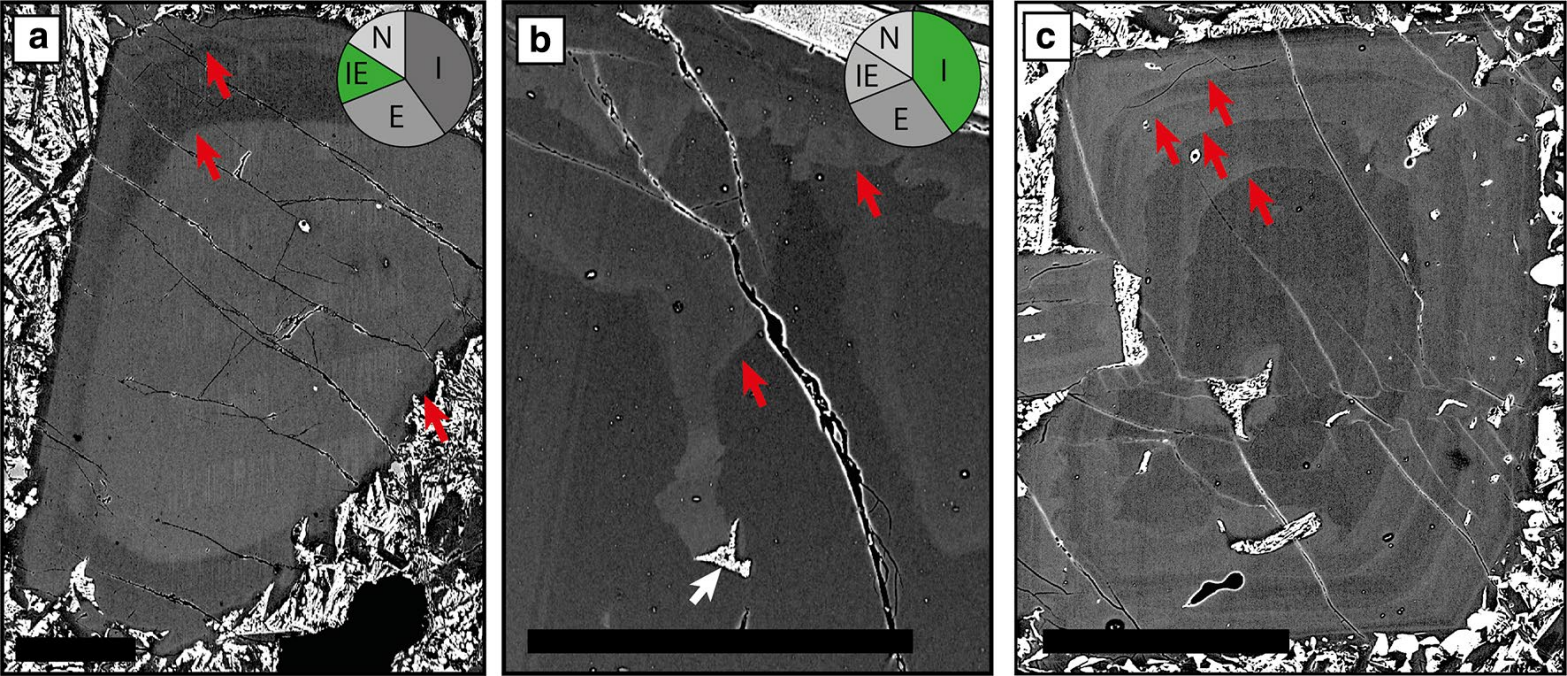
Types of plagioclase resorption. **a** Plagioclase showing both internal and external resorption events. **b** Plagioclase with two internal resorption events. **c** Plagioclase with multiple internal resorption events. Red arrows indicate the position of resorption interfaces within the plagioclase crystals. White arrows indicate melt inclusions. Pie charts illustrate the proportions of each type of resorption in all individual plagioclase crystals in the database. Pie chart abbreviations are as follows: *E* external, *IE* internal and external, *N* no resorption, *I* internal. All scale bars are 0.25 mm

84% of individual plagioclase crystals show evidence of resorption. This resorption is expressed in several ways including resorbed crystal habits (i.e. external resorption) (Figs. 4b, 9a) and resorption interfaces within the plagioclase crystals (i.e. internal resorption) (Figs. 6c, 7a, b, d, 9a–c). Figure 9a shows that some plagioclase have both internal and external resorption; internal resorption is the most common type of resorption (Fig. 9b; Table S7). Zoning outboard of internal resorption events is more often reverse (63%) than normal (37%); some resorption events are followed by thin normally zoned bands that may or may not be melt inclusion rich. Tabular and resorbed crystals show more evidence of resorption (e.g. average number of events per crystal and total number of events) compared to skeletal and acicular crystals (Table S8); there is no relationship between crystal area and number of resorption events (Fig. S3). Amoeboid melt inclusion are often found in plagioclase that show evidence of resorption (Fig. 8c, e); they are the largest type of melt inclusion (Fig. S4, Table S9). Melt inclusions can also be associated with internal resorption interfaces (e.g. Figs. 4a, 9b). Similar to individual crystals, resorption in monomineralic glomerocrysts is most commonly internal (e.g. Fig. 6c), with 81% recording at least one event. Whilst the location and number of resorption events were not recorded in polymineralic glomerocryst components, 55% of these glomerocrysts show evidence of resorption.

#### Glomerocryst configuration and component contacts

Glomerocrysts are found in both open (Fig. 6a, b) and closed configurations (Fig. 6c). Closed configurations are more common in monomineralic glomerocrysts, whilst both configurations are as common in polymineralic glomerocrysts (Table S10). Embedded component contacts are the most common in all glomerocrysts, while planar (Fig. 6c) and point contacts (Fig. 6a, b) are more common in mono- and polymineralic glomerocrysts, respectively (Table S10). Glomerocrysts commonly show more than one component contact type.

### Plagioclase major element compositions

**Fig. 10:**
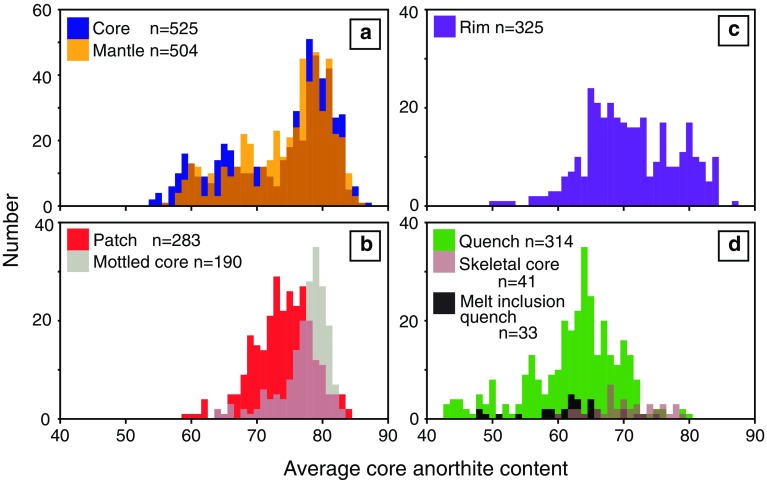
Anorthite (defined as Ca/(Ca + Na) × 100) content of all plagioclase analyses separated by analysed crystal location (e.g. core, rim etc.). **a** Plagioclase cores and mantles; mantles are defined as intervening regions of the crystal between a clearly defined core and rim. **b** Plagioclase mottled cores and patches. **c** Plagioclase rims. **d** Plagioclase skeletal cores, quench and melt inclusion rims. There are few relationships between analysed location and composition. Note: for all crystal locations, other than those in **b,** each value is an average of multiple analyses; values in **b** are not averages due to the heterogeneous nature of both patches and mottled cores; therefore the number of analyses does not reflect the number of plagioclase analysed

Plagioclase major element compositions are presented in Online Resource 1. The An content of plagioclase core (An_54–87_) and mantle (An_56–86_) compositions show multimodal distributions with peaks at ~ An_80_, ~ An_68_ and ~ An_60_; the two lower An core peaks are shifted towards lower An compared to mantle peaks (Fig. 10a). Patches (An_59–84_) and mottled cores (cores that show diffuse patchy zoning, Fig. 8g) (An_64–83_) show peaks in compositions at ~ An_75_ and ~ An_79_, respectively (Fig. 10b), similar to cores and mantles. Plagioclase rim compositions (An_50–87_) show at least two broad compositional peaks at ~ An_70_ and ~ An_80_ (Fig. 10c). Quench rim compositions (An_43–80_) define a multimodal distribution with a main compositional peak located at ~ An_64_; melt inclusion quench rims (An_48–75_) and skeletal core (An_60–79_) compositions fall within the range defined by quench rims (Fig. 10d). The range of average core An contents of megacrysts (An_54–87_), microcrysts (An_54–85_) and monomineralic glomerocrysts (An_58–85_) are similar to one another and differ to the more restricted range of polymineralic glomerocrysts (An_54–79_) (Fig. S5).

Crystal habit is correlated with An content: tabular and resorbed plagioclases have higher average core An contents than acicular, skeletal and cores of matured skeletal crystals (Fig. 5a). Poly- and monomineralic glomerocrysts have similar size ranges; however the core An content of polymineralic glomerocryst components is lower than the monomineralic glomerocryst components (Fig. 5b); the latter have core An contents similar to tabular and resorbed crystals.

### Plagioclase–melt equilibria

Of the 34 basaltic glasses analysed in Lissenberg et al. (2019), only 18 samples contain both glass and plagioclase, enabling the calculation of plagioclase–melt equilibria. To these we added additional glass analyses from van der Zwan et al. (2017) (HLY0102-D12-1 and HLY0102-D21-4) and Gale et al. (2013) (HLY0102-D18-1, HLY0102-D18-6, HLY0102-D22-1, HLY0102-D21-1, HLY0102-D23-36, HLY0102-D26-6, HLY0102-D27-32 and HLY0102-D66-32), for which plagioclase data are presented herein. We used the model of Grove et al. (1992) to calculate plagioclase–melt equilibria in MORB:

1 $${\text{An}} = \frac{A}{(1 + A)},$$

where *A* is defined as:

2 $$\begin{aligned} A & = ~\frac{{X_{{{\text{CaAl}}_{2} {\text{O}}_{4} ^{{{\text{Liq}}}} }} }}{{X_{{{\text{NaAlO}}_{2} ^{{{\text{Liq}}}} }} ~ \times ~X_{{{\text{SiO}}_{2} ^{{{\text{Liq}}}} }} }} \times {\text{EXP}}~\Bigg[ {11.1068 - 0.0338~ \times P\,({\text{in~kbars}}) - 4.4719} \\ & \quad { \times ~\left( {1 - X_{{{\text{NaAlO}}_{2} ^{{{\text{Liq}}}} }} } \right)^{2} - 6.9707~ \times ~\left( {1 - X_{{{\text{KAlO}}_{2} ^{{{\text{Liq}}}} }} } \right)^{2} } \Bigg]. \\ \end{aligned}$$

**Fig. 11:**
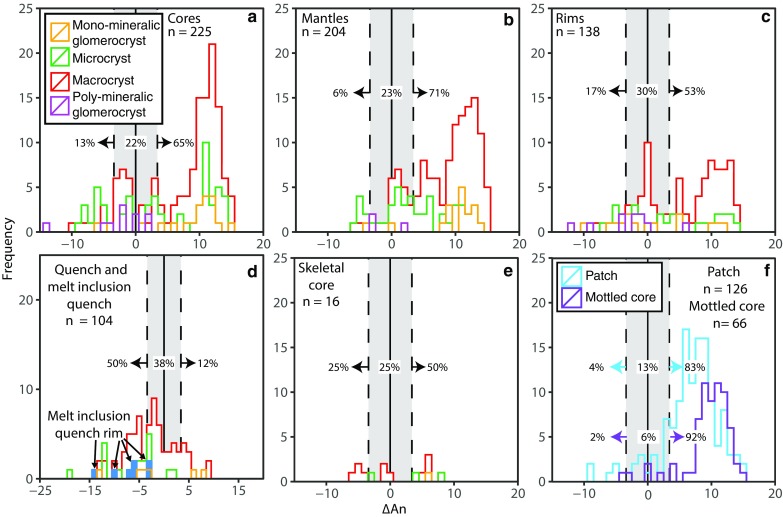
Histograms of ΔAn for each crystal location split by crystal type. **a** Cores. **b** Mantles. **c** Rims. **d** Quench and melt inclusion quench rims. **e** Skeletal cores. **f** Patches and mottled cores. Whilst some analyses plot in equilibrium with their host melts (within the grey area), a vast proportion of analyses plot in equilibrium with more primitive melt compositions. Percentages on each panel represent the percentage of analyses that plot in equilibrium with the melt or with more evolved (left) or primitive (right) melts. Solid black vertical lines represent equilibrium (ΔAn = 0); dashed lines represent ± 5% error (Grove et al. 1992) on the average plagioclase anorthite content (An_69_) calculated using Eqs. (1) and (2). ΔAn = An_measured_ − An_calculated_. An_measured_ is the An content determined from EDS analysis, An_calculated_ is the An content calculated from Eqs. (1) and (2)

These equations were calibrated on 171 plagioclase–liquid assemblages produced experimentally at pressures from 0.001 to 27 kbar, and predict the equilibrium plagioclase composition as a function of both pressure and melt composition (Grove et al. 1992). The equations use the liquid albite, anorthite and orthoclase components of selected Gakkel Ridge glasses calculated following the equations of Bottinga and Weill (1972). Equilibrium plagioclase compositions have been calculated assuming a pressure of 2 kbar; because An contents of plagioclase decrease by only ~ 1% An per kbar, and pressures at the Gakkel Ridge are not expected to be larger than ~ 3 kbar (Shaw et al. 2010); the assumed pressure has little effect on the overall results. Our calculations show that many of the crystals are not in equilibrium with their host glasses (Fig. 11). We define the degree of disequilibrium as ΔAn (An_measured_ − An_calculated_): hence, equilibrium is at ΔAn = 0. For example, only 22% of crystal cores are in equilibrium with their host melts, 13% and 65% plotting in equilibrium with more evolved and primitive melts, respectively (Fig. 11a). Plagioclase rims (Fig. 11c), quench rims and melt inclusion quench rims (Fig. 11d) show higher percentages in equilibrium with their host melts; however, 53% of rim analysis still plot in equilibrium with more primitive melts.

Values of ΔAn for each crystal location analysed (e.g. core, rim) overlap (Fig. 11). Plagioclase cores, mantles, rims and patches all have similar ΔAn ranging from − 14 to + 15; mottled cores range from − 4 to + 15. Cores, mantles, rims and patches and mottled cores show peaks around ΔAn = 12 and ΔAn = 9, respectively. Skeletal cores straddle equilibrium with more restricted ΔAn values of − 6 to + 8. Quench and melt inclusion quench rims have the most extreme negative ΔAn (≤ − 19). Several relationships between ΔAn and crystal type are present in Fig. 11: (1) macrocrysts and monomineralic glomerocrysts show similar distributions; (2) microcrysts show a similar distribution to both macrocrysts and monomineralic glomerocrysts, but can extend to lower ΔAn; and (3) with the exception of one rim analysis, polymineralic glomerocrysts tend to plot within equilibrium or towards lower ΔAn.

### Plagioclase TiO_2_ content

**Fig. 12:**
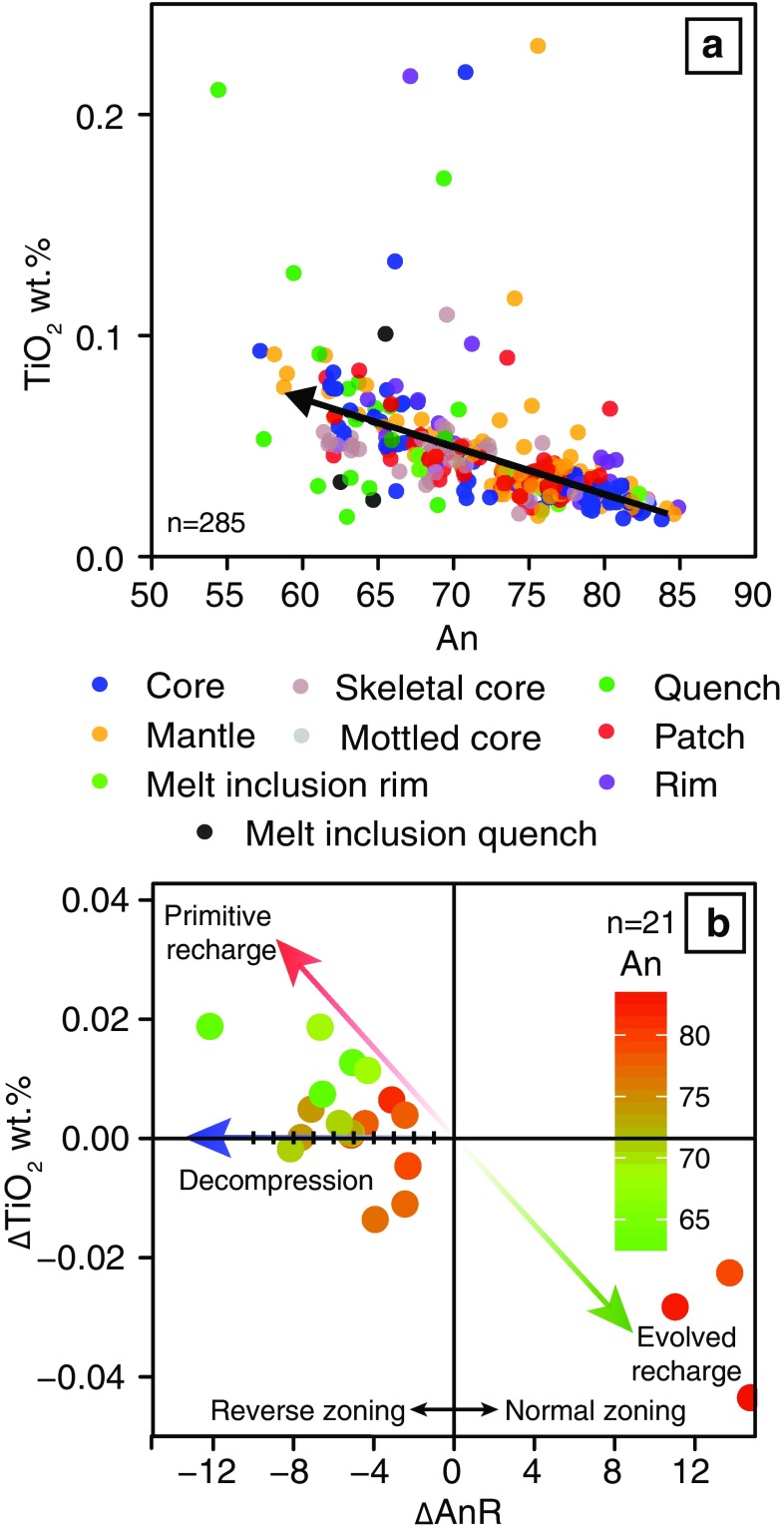
**a** Plagioclase TiO_2_ and An contents correlate negatively. There is no correlation between plagioclase TiO_2_ content and location (e.g. core, rim etc.). **b** Relationship between ΔAnR and ΔTiO_2_. Analyses plot both along the decompression vector and within quadrants for both primitive and evolved recharge, suggesting resorption may occur due to both decompression and magma mixing. Intervals along the decompression vector represent 1% An steps

Plagioclase TiO_2_ contents range from 0.02 to 0.34 wt% (Online Resource 1). There are no clear relationships between crystal location and TiO_2_; TiO_2_ contents of all crystal locations overlap (Fig. 12a). However, TiO_2_ contents correlate negatively with plagioclase An content (Fig. 12a). Two parameters, ΔTiO_2_ and ΔAnR, have been calculated here to quantify the change in composition across a resorption interface. These are defined as TiO_2inboard_ − TiO_2outboard_ and An_inboard_ − An_outboard_, respectively. Plagioclase plot from + ΔAnR and − ΔTiO_2_ to − ΔAnR and + ΔTiO_2_ (Fig. 12b).

### Juxtaposition of chemically and texturally distinct populations

**Fig. 13:**
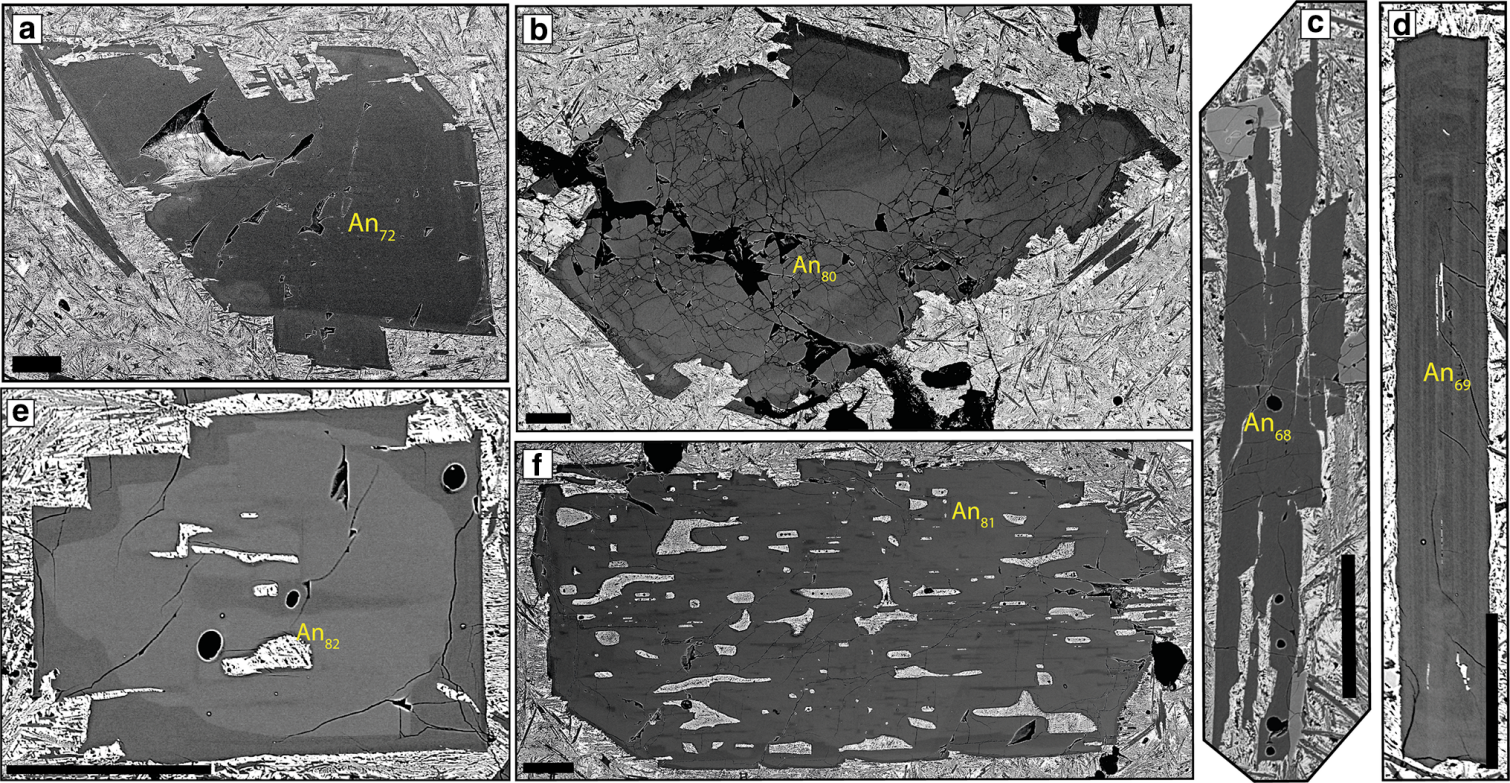
An example of juxtaposition of chemically and texturally distinct plagioclase crystals within sample HLY0102-D22-1. Crystal habits include resorbed (**a**, **b**, **f**), skeletal (**c**) and tabular (**d**, **e**). Zoning can be simple (oscillatory **d**) or show more complex forms (patchy zoning **e**, **f**). Average core anorthite contents in this sample range from An_68_ to An_82_. All scale bars are 0.25 mm.

Individual samples contain multiple distinct chemical and textural populations (Fig. 13). Considering the maximum An content of cores, 73% of samples show a range equal to or greater than 5% An with an average range of 10% An; the smallest range is 1%. Texturally, zoning complexity within a single sample ranges from 0 to 10, and 80% of samples show zoning complexity ranges greater than 3; only three samples possess plagioclase crystals with no variation in zoning complexity. Although the variation in core An content could in part relate to sectioning effects, this variation agrees well with the observed textural variation. For example, plagioclases in Fig. 13c, d have very similar core anorthite content but exhibit both different zoning patterns and crystal habits (no zoning and skeletal habit Fig. 13c and oscillatory zoning and tabular habit Fig. 13d).

## Discussion

We have shown that plagioclase crystal cargo from the Gakkel Ridge is complex and records a range of textures and compositions. Below we shall discuss how these textures and compositions relate to magmatic processes.

### Significance of plagioclase textures

#### Skeletal growth: disequilibrium during undercooling

A first-order observation that can be taken from the presented plagioclase compositions is that skeletal crystals and skeletal cores of matured skeletal crystals have lower core An contents compared to tabular and resorbed habits (Fig. 5a). Whilst this could be interpreted as the different crystal habits having grown under different pressure or temperature conditions or from melts of different bulk compositions, their morphologies and more sodic compositions are better explained as the result of their growth mechanism. It has been shown experimentally that under strong undercooling, plagioclase growth is diffusion limited and results in plagioclase crystals that are more sodic than those that would grow under equilibrium conditions (Lofgren 1974). Sodic growth during strong undercooling is demonstrated well by crystal and melt inclusion quench rims which have more sodic compositions (Fig. 10d) and negative ΔAn values (Fig. 11d). In contrast, when the system is not strongly undercooled, growth from a melt is interface controlled (Kirkpatrick et al. 1979) resulting in the growth of the remaining non-skeletal crystal cargo.

An important observation is that matured skeletal crystals record periods of undercooling distinct from that occurring during quench crystallisation (e.g. crystallisation of quench rims and some skeletal plagioclase), suggesting multiple periods of disequilibrium and undercooling within the magmatic system. Both magma mixing (e.g. Kuo and Kirkpatrick 1982) and intrusion of melts into cold regions of the plumbing system (e.g. Meyer and Shibata 1990; Hellevang and Pedersen 2008) have been attributed to undercooling and the formation of similar skeletal plagioclase from the Mid-Atlantic, Mohns and Knipovich Ridges. To our knowledge, similar matured skeletal plagioclase has not been reported from fast-spreading ridges. This cannot be due to a lack of magma mixing, as there is abundant evidence suggesting the importance of this process (Dungan et al. 1978; Grove et al. 1992; Pan and Batiza 2003; Ridley et al. 2006). Instead, the thinner and warmer lithosphere at fast-spreading ridges may reduce the magnitude of undercooling experienced by magmas as they move through the magmatic system. Alternatively, matured skeletal plagioclase at fast-spreading ridges may simply not be erupted. This is supported by a recent study by Lange et al. (2013) that suggests axial melt lenses act as density filters. They propose that when melts enter axial melt lenses at fast-spreading ridges, the ascent velocity drops sufficiently to cause plagioclase to settle out of its host liquid and remain within the magmatic system. Whilst we cannot definitively say which factor led to the undercooling recorded by matured skeletal plagioclase at the Gakkel Ridge, the presence of a cool lithosphere likely favours the occurrence of undercooling as melts advance through the magmatic system; the absence of a steady-state plumbing system such as that at fast-spreading ridges favours their eruption. The presence of thin normally zoned melt inclusion-rich zones within plagioclase may provide additional evidence for this process as suggested by Hellevang and Pedersen (2008).

#### Resorption: recharge and decompression

Crystal resorption can occur through multiple processes, as illustrated in Fig. 2. Whilst, as indicted by the blue arrow in Fig. 2a, heating alone can cause resorption, we observe resorption interfaces associated with changes in compositions (Figs. 7, 8, 9) suggesting that the process(s) causing resorption must involve a change in composition of the system. The process(s) must also occur repetitively to explain the presence of multiple resorption interfaces within individual plagioclase (e.g. Fig. 9). Magma mixing is one mechanism that can cause changes in both the temperature and bulk composition of the system and is often invoked to explain resorption observed in mid-ocean ridge systems (e.g. Hellevang and Pedersen 2008). For example, during magma mixing, pre-existing plagioclase (*X*_2_) is superheated (*X*_2_–$X_{2}^{\prime }$) and becomes resorbed, as the system tends towards equilibrium, and if the plagioclase was not completely dissolved, reverse zoning can form ($X_{2}^{\prime }$–*X*_4_) (Fig. 2b). Alternatively, Fig. 2a shows that a reduction in pressure (yellow–green) causes the equilibrium plagioclase composition to become more anorthitic, thus causing pre-existing plagioclase to become unstable; if the plagioclase is not completely dissolved, subsequent crystallisation is more anorthitic and reverse zoning forms.

Both magma mixing and decompression could occur multiple times within the system. However, because both of these processes result in similar textures and changes in major element composition, these cannot be used alone to distinguish the cause of resorption. Here, we use plagioclase TiO_2_ contents to distinguish between magma mixing- and decompression-induced resorption. The underlying rationale is that TiO_2_ and An content form a well-defined negative correlation (Fig. 12a). In principle, this correlation can be related to (1) an increase in TiO_2_ concentration in the melt during magmatic differentiation and (2) an increase in the Ti partition coefficient with both decreasing An content of the crystallising plagioclase and evolution of the melt composition (Bédard 2005). However, over the range of An_60–85_ the Ti partition coefficient only increases by a factor of 1.5 (Eq. 8 of Bédard 2005) compared to TiO_2_ that increases by a factor or 4. The change in partition coefficient is therefore not sufficient to explain the observed negative correlation; hence, we interpret the An–TiO_2_ correlation (Fig. 12a) to reflect a liquid line of decent. Assuming crystallisation occurs under equilibrium conditions, crystallisation following mafic recharge would have higher An and lower TiO_2_ contents than crystallisation from a more evolved magma. Secondly, plagioclase TiO_2_ contents are relatively insensitive to changes in pressure with the partition coefficient of Ti in plagioclase changing by 0.01/GPa (Bédard 2005). Therefore, decompression-induced resorption would result in subsequent crystallisation of plagioclase that was reversely zoned, but that has TiO_2_ contents the same as the adjacent compositional zone formed prior to resorption.

We observe three relationships in Fig. 12b that suggest both decompression- and magma mixing-induced resorption have occurred. Magma mixing, involving evolved and primitive recharge, is supported by normal and reverse zoning outside of the resorption interface that have positive and negative ΔTiO_2_ values, respectively (Fig. 12b). Those resorption interfaces that show reverse zoning in An and no change in TiO_2_ suggest that resorption has resulted from decompression (Fig. 12b). The ΔAn across these interfaces ranges from ~ − 4 to − 8, which, assuming the pressure dependence of plagioclase is ~ 1.3% An/kbar (see Online Resource 2), corresponds to decompression over ~ 9–18 km. Whilst a single decompression interval over these depths may seem high, the lithosphere at the Gakkel has been estimated to be up to 35 km thick (Schlindwein and Schmid 2016), indicating that there is ample distance over which decompression could occur. In addition, new plagioclase-hosted melt inclusion data from the Gakkel Ridge extend the melt inclusion crystallisation pressure record to ~ 16 km (Bennett et al. 2019). Therefore, the depth range inferred from the plagioclase anorthite contents is consistent with independent constraints on the lithospheric thickness and depths of crystallisation

Whilst normal zoning and negative ΔTiO_2_ can indicate evolved recharge, it may also suggest crystallisation under disequilibrium conditions. During disequilibrium, TiO_2_ can become concentrated in a chemical boundary layer at the melt–crystal interface (Bottinga et al. 1966). Continued crystal growth may ultimately cause TiO_2_ to become incorporated into the plagioclase resulting in both normal zoning and negative ΔTiO_2_ values. However, (1) only one of the three plagioclase crystals in this field (the lowest ΔTiO_2_ and highest ΔAnR in Fig. 12b) shows morphological evidence of skeletal growth (e.g. outer rim that shows some intergrowth with the groundmass) and (2) the absolute TiO_2_ contents of the outboard portions of these crystals are not elevated at low An as expected during skeletal growth; we therefore propose that at least two of the analysed crystals have experienced resorption associated with evolved recharge.

Regardless of whether normal zoning and negative ΔTiO_2_ reflect evolved recharge or disequilibrium growth, reverse zoning is the most common type of zoning following resorption. This suggests that the processes of primitive recharge and/or decompression are more common.

#### Patchy zoning: infilling following skeletal growth and resorption

Patchy zoning is often attributed to two different processes: (1) resorption and subsequent overgrowth (Vance 1965; Maaløe 1976; Ginibre and Wörner 2007; Ridley et al. 2006) and (2) skeletal growth and subsequent infilling (Kuo and Kirkpatrick 1982; Meyer and Shibata 1990). Patchy zoning observed here exhibits two morphologically distinct end members, geometric (Type 1) and amoeboid (Type 2) that we suggest result from the two processes.

Type 1 patchy zoning is geometric and present in matured skeletal crystals that have skeletal cores (Fig. 8a, b). These crystals are commonly reversely zoned and have high An patches within the skeletal core, or low An patches within the reversely zoned region (Fig. 8a, b). To understand the origin of Type 1 zoning, we first need to determine the origin of the reverse zoning. The lack of resorption at the junction between the skeletal core and surrounding high An region suggests the reverse zoning is not related to changes in magma composition or pressure, and instead likely reflects changes in growth kinetics. Crystallisation experiments of Lofgren (1974) have produced reverse zoning in skeletal plagioclase crystals under conditions of strong undercooling as the result of the crystal attempting to re-attain equilibrium growth conditions (Smith and Lofgren 1979); it is through this mechanism that we suggest reverse zoning within these crystals has formed. We propose a two-stage origin for Type 1 patchy zoning (Fig. 2b, d) whereby initial undercooling (∆*T*_U_) within the magmatic system forms lower An geometric skeletal core (*S*_1_ in Fig. 2b, d). This core-forming stage is followed by a period when the crystal attempts to re-attain equilibrium (*S*_1_–*S*) resulting in overgrowth and infilling of the skeletal core with high An plagioclase producing matured skeletal crystals (*S*_2_). The low and high An patches we observe are a result of sectioning of the matured skeletal plagioclase crystal. Isolated patches of low An correspond to the skeletal core; in 3D these isolated patches would be connected to the remaining skeletal crystal. High An patches are the result of infilling of voids within the original skeletal framework.

Type 2 patchy zoning is characteristically amoeboid in form (Fig. 8c–g). We interpret the irregular shape of Type 2 patches as the result of crystal resorption (Fig. 2c, *X*_2_–$X_{2}^{\prime }$) that either proceeded along cleavage planes, resulting in a degree of crystallographic alignment and elongation of patches (Fig. 8c) or occurred randomly throughout (Fig. 8d–g). Patches are often associated with amoeboid melt inclusions that possess zonation about their margins; this zoning can be similar to zoning around the outside of the host crystal (Fig. 8f). This suggests that melt inclusions have not been occluded and have remained connected to the matrix following resorption. Any subsequent crystallisation occurs on both the exterior of the crystal and walls of the inclusions (Fig. 2c, $X_{2}^{\prime }$–*X*_4_). Depending on where the crystal is sectioned, one may observe a patch or a melt inclusion with a rim (Fig. S6). Our interpretation that voids formed during resorption remained connected to the matrix is supported by the similar compositions of isolated patches (i.e. those not associated with melt inclusions) and other compositional zones of the crystal (Fig. 8c). We do not consider zoned patches to have formed from the crystallisation of trapped melt as suggested by Vance (1965) and Meyer and Shibata (1990), due to the absence of multi-phase inclusions associated with isolated patches.

#### Glomerocrysts: mush fragments and synneusis

Plagioclase crystal cargo contains both poly- and monomineralic glomerocrysts (Fig. 6a, b). We interpret poly- and monomineralic glomerocrysts that have open structures and plagioclase components at high angles to one another (Fig. 6a, b) as portions of crystal mush networks that have been entrained into an ascending melt. High-angle, predominantly plagioclase networks have high porosities and have previously been interpreted as pieces of immature crystalline mush (e.g. Holness et al. 2005). Similar crystal networks from the East Pacific Rise are interpreted as pieces of entrained crystal mush (Pan and Batiza 2003; Moore et al. 2014). The presence of olivine within some of the polymineralic glomerocrysts (e.g. Fig. 6a) suggests that in some instances, olivine may remain stuck within the mush zone. In contrast to those with open mush network structures, some glomerocrysts have closed structures with components at low angles to one another (Fig. 6c). Whilst the lack of open structures and presence of planar contacts may reflect derivation from a more compacted portion of the mush system, they could equally have formed through synneusis. Synneusis is the drifting together and attachment of like phases during turbulent flow (Vance and Gilreath 1967) and is likely to occur during melt ascent. It might be that individual glomerocryst components originate from a mush zone, and that following mush zone disaggregation the components may come together through synneusis during transport in the melt.

Whilst polymineralic glomerocrysts can exhibit simpler zoning than monomineralic glomerocrysts, components in each record evidence of complex crystallisation histories (e.g. resorption, patchy zoning and complex zoning combinations), with more evolved compositions of some glomerocrysts suggesting they were derived from more evolved regions of the plumbing system. Indeed, the presence of plagioclase rims in both poly- and monomineralic glomerocrysts that are too evolved to be in equilibrium with their host melts indicates that the glomerocrysts equilibrated in an evolved mush zone before being picked up by a more primitive host melt (Fig. 11c). Reverse zoning in 20% of the 45 monomineralic glomerocrysts components that had both cores and rims analysed further indicates that mush zones experienced primitive recharge, with some plagioclase growing directly from the recharging melt. In addition to the evidence of primitive recharge, rims of some glomerocrysts that are too primitive to be in equilibrium with their host melts (Fig. 11c) indicate that they were entrained by more evolved melts. Taken together, these lines of evidence demonstrate that both primitive and evolved mush zones are present in the Gakkel Ridge and that mush zone components were entrained by both primitive and evolved recharging melts.

### Melt inclusion entrapment

We observe a relationship between melt inclusion morphology and host plagioclase textures that suggests different morphologies result from different processes; understanding how these different morphologies form is necessary to correctly interpret melt inclusion data (Michael et al. 2002; Faure and Schiano 2005). Within matured skeletal crystals, boxy, often elongated melt inclusions occur in association with both reversely zoned portions of the crystals and geometric high An patches within the low An skeletal core (Fig. 8a, b). We propose that these melt inclusions originate as hollows with the geometric skeletal core. Infilling of the voids by reversely zoned plagioclase suggests that the voids formed during skeletal growth were not immediately isolated from the matrix. Continued connection to the matrix means that any modification to the melt composition caused by the initial skeletal growth (i.e. plagioclase incompatible element enrichment in adjacent boundary layer (Bottinga et al. 1966)) can become dissipated over time if element diffusivities are rapid enough (Danyushevsky et al. 2002). If at a later stage these voids become isolated from the matrix, the composition of the inclusion would not be the same that existed at the time of initial skeletal growth, nor would it record geochemical evidence of skeletal growth. Only if melt inclusions were occluded rapidly would chemical evidence of skeletal growth be observed.

Similarly, resorption causes melt adjacent to resorbed plagioclase to become enriched in plagioclase components (Nakamura and Shimatika 1998; Danyushevsky et al. 2002); melt inclusions will record these modified compositions if melt inclusions are occluded from the matrix soon after the initial resorption event. In the Gakkel samples, Type 2 patchy zoning and marginal zoning around melt inclusions suggest that occlusion did not occur rapidly, and inclusions remained connected to the matrix. Therefore, as with the melt inclusions in the skeletal crystals, the composition of resorption-related amoeboid melt inclusions may not record the melt composition at the time of resorption or any compositional modification resulting from initial resorption. These amoeboid melt inclusions are found at multiple levels within plagioclase; around the margins, associated with internal resorption interfaces (Figs. 4a, 9b) or randomly distributed throughout (Fig. 8e). Where melt inclusions are related to resorption interfaces, it may be possible to determine a relative timing for their formation. However, Cashman and Blundy (2013) have highlighted that the three-dimensional nature of melt inclusions and their prolonged connection to the matrix mean that there is often no spatial pattern to volatile contents and in turn entrapment pressures; this should be kept in mind when determining the evolutionary stages of a system.

The association of particular melt inclusion morphologies with specific textures in plagioclase demonstrates that their formation may be associated with processes which alter the composition of trapped melts. If boundary layers do become trapped within melt inclusions, compositions of these inclusions will not faithfully represent the true composition of melts within the magmatic system. Equally, if melt inclusions remain connected to the matrix for a period before occlusion, their compositions will not relate to those that existed when the inclusion formed. Therefore, without a thorough understanding of how melt inclusions formed, melt inclusion compositions cannot be reliably used as tools to study the compositional variability of magmatic systems.

### The origin of plagioclase crystal cargo

Within mid-ocean ridges, axial melt lenses, dykes, and mush zones are all potential sources of the plagioclase crystal cargo. When considering the origin of plagioclase in the Gakkel Ridge, any model needs to account for the following observations: (1) mixed crystal populations; (2) crystal content; (3) open-structured glomerocrysts; (4) plagioclase–melt disequilibrium; (5) plagioclase habits formed indicative of undercooling; and (6) multiple periods of resorption. Below, we shall explore whether these observations are consistent with plagioclase crystal cargo originating from a melt (i.e. axial melt lens or dykes) or crystal (i.e. mush zone) dominated system.

The presence of plagioclase with contrasting magmatic histories within individual samples (Fig. 13) requires an efficient mechanism by which these crystals become juxtaposed. Convection within a melt body, such as an axial melt lens, is one mechanism by which this could occur, and would need two criteria to be met: (1) the presence of a magma chamber; and (2) low enough magma crystallinities to facilitate convection. To date, melt lenses at multiple depths have been identified at fast- (e.g. Detrick et al. 1987; Marjanović et al. 2014) and intermediate-spreading (e.g. Canales et al. 2005) ridges, with similar, likely ephemeral lenses identified along limited portions of slow-spreading ridges (e.g. Sinha et al. 1998; Singh et al. 2006). Melt lenses are often segmented into crystal-rich and crystal-poor regions (Singh et al. 1998; Xu et al. 2014; Marjanović et al. 2015); melt within melt-dominated regions has low viscosities and can convect freely (e.g. Sinton and Detrick 1992). Whilst an axial magma reservoir containing 3–10% melt was recently identified beneath the ultraslow-spreading Southwest Indian Ridge (Jian et al. 2017), there is as yet no evidence to support the presence of melt lenses similar to those at fast-spreading ridges beneath ultraslow-spreading ridges, including Gakkel. Additionally, as magma crystallinity reaches 20–25%, magma viscosity increases to the point where the system is essentially a mush (Marsh 1989) and convection is greatly inhibited (Sinton and Detrick 1992). Basalt samples reported here have crystal contents up to 50% (Fig. 3). Therefore, both the lack of melt lenses along the Gakkel Ridge and high crystal content of many basalts are inconsistent with crystal cargo originating from a melt-rich body. Nonetheless, the low crystal content of some basalts (Fig. 3) indicates that melt-dominated regions may exist locally within the Gakkel Ridge plumbing system.

As opposed to crystallisation in a melt lens, the plagioclase crystal cargo may have crystallised during dyke injection (e.g. Zellmer et al. 2011). However, multiple observations are at odds with this relatively simple model and instead suggest that crystallisation during magma ascent was limited. Firstly, if crystallisation occurred solely during dyke ascent, one would expect either equilibrium between the host glass and crystals, or disequilibrium growth of low An crystals if undercooling prevails. However, the data suggest that much of the plagioclase crystal cargo is in equilibrium with melts that are more primitive than the host glasses (Fig. 11). Of note is the observation that plagioclase crystals in samples from the same dredge analysed by Zellmer et al. (2011) show some of the most extensive disequilibrium (Fig. S7). Furthermore, the zoning complexity of the Gakkel plagioclase is typically high (Fig. 7), indicating that multi-stage histories, often including resorption (Fig. 9), are the norm. However, lower zoning complexity values and number of resorption events recorded by skeletal and acicular plagioclase (Tables S3, S8) indicate they experienced simpler histories than resorbed and tabular forms which may relate to the timing of crystallisation (e.g. skeletal and acicular plagioclase have grown during late periods of undercooling). Finally, the juxtaposition of chemically and texturally distinct plagioclase populations within samples (Fig. 13) indicates that individual crystals experienced different magmatic histories and, with the exception of the few true phenocrysts and micro-phenocrysts, were likely entrained into an ascending melt from distinct parts of the plumbing system.

Alternatively, crystal cargo could have originated from a crystal-dominated region within the plumbing system where melt viscosities are low and crystallinities are high (Sinton and Detrick 1992). The presence of mush zones at mid-ocean ridges is supported by both geophysical (e.g. Singh et al. 1998; Crawford et al. 1999) and petrological (e.g. Pan and Batiza 2003; Ridley et al. 2006) evidence, whilst their disaggregation has been identified as a key process occurring in basaltic systems (Sinton and Detrick 1992; Hansen and Grönvold 2000; Pan and Batiza 2003; Costa et al. 2009; Passmore et al. 2012; Lange et al. 2013; Neave et al. 2014, 2017). Multiple observations support that this process plays a vital role in the origin of the plagioclase crystal cargo at the Gakkel Ridge.

Firstly, the high crystal content of some samples is more consistent with an origin from a mush zone as opposed to a convecting melt body. Whilst one might expect other phases that might have formed in the mush zone, such as olivine, to be present in erupted crystal cargo, we observe an enrichment in plagioclase relative to olivine as the total crystal contents increases (Fig. 3). This plagioclase enrichment has been identified in other MORB crystal cargo (e.g. Bryan 1983) and is a common feature of PUBs sampled at intermediate- to ultraslow-spreading ridges (e.g. Lange et al. 2013). This enrichment may reflect the loss of olivine by gravity settling during melt ascent following mush disaggregation or, as suggested by Lange et al. (2013) for the origin of PUBs, the disruption of non-cotectic plagioclase-rich cumulates. Secondly, whilst individual plagioclase crystals might represent parts of disaggregated mush, glomerocrysts, in particular those with open crystal networks interpreted as pieces of entrained crystal mush, support an origin for at least some of the crystal cargo through mush disaggregation. Finally, the amount of disequilibrium (Fig. 11) and physical evidence for resorption (Fig. 9) support the idea that plagioclase crystal cargo did not grow from its host melt and was entrained from elsewhere. Instead, as suggested by diffusion studies (Costa et al. 2009; Moore et al. 2014), we propose that pre-existing mush zones become disaggregated following melt replenishment; plagioclase from these mush zones is subsequently out of equilibrium with the host melt. Through disaggregation, plagioclase from different parts of the mush zone that have experienced different magmatic histories becomes juxtaposed, resulting in mixed chemical and textural populations (e.g. Fig. 13). It is important to highlight that multiple resorption events within single plagioclase crystals indicate that the process of melt replenishment and mush disaggregation may occur multiple times within the magma plumbing system. Alternatively, following disaggregation, melts with their entrained crystal cargo may experience staged decompression resulting in multiple periods of disequilibrium and subsequent resorption.

## Synthesis

**Fig. 14:**
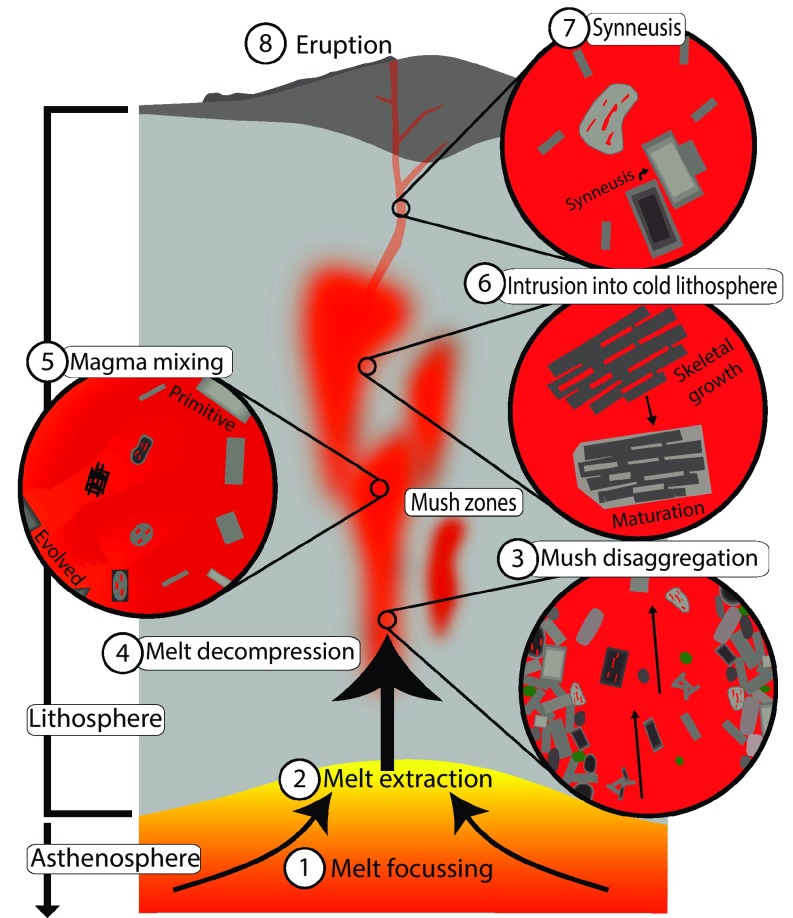
Schematic model for the processes occurring within the Gakkel Ridge plumbing system. The Gakkel Ridge comprises the asthenosphere overlain by lithosphere comprising a basaltic cap directly overlying mantle peridotite. Processes within the Gakkel Ridge are as follows: 1 melt focussing, 2 melt extraction, 3 mush disaggregation, 4 melt decompression, 5 magma mixing, 6 intrusion into cold lithosphere, 7 synneusis, and 8 eruption. See text for “Discussion”. Note: all processes other than 1, 2 and 8 can occur in any order

The textural and compositional complexity of the plagioclase crystal cargo presented here allows us to reconstruct the processes occurring within the plumbing system of the ultraslow-spreading Gakkel Ridge (Fig. 14). Following initial melt focussing (1) and extraction (2) from the asthenosphere, melts are intruded into the lithosphere that comprises a relatively thin basaltic crust directly overlying mantle peridotite. Intrusion into the mantle lithosphere and subsequent crystallisation produce a mushy, crystal-dominated plumbing system within which variable degrees of fractionation can occur. Within these zones, there may locally be melt-rich regions as suggested by the low crystal contents of some samples. Multiple lines of evidence suggest that later melt replenishment, with both primitive and evolved melts, causes mush zones to become disaggregated (3), with mush components becoming incorporated into the ascending melt in the form of both individual crystals and networks. Disaggregation of different parts of the mush zone results in the juxtaposition of texturally and chemically distinct plagioclase populations. The simpler zoning complexities of skeletal and acicular plagioclase indicate they had simpler crystallisation histories compared to tabular and resorbed forms and may have formed during later periods of undercooling. Repeated episodes of melt decompression (4) and magma mixing (5) may generate periods of disequilibrium and resorption. Periods of crystallisation following initial resorption generate Type 2 patchy zoning. Evidence for periods of disequilibrium growth is also present in the form of skeletal and matured skeletal crystals. Matured skeletal crystals result from periods of undercooling that are distinct to that occurring upon quench crystallisation and could result from both magma mixing (5) and intrusion into cool regions of the lithosphere (6). Subsequent re-attainment of equilibrium growth mechanisms results in the generation of Type 1 patchy zoning and matured skeletal crystals. The presence of repeated reverse zones separated by thin normal zones that may or may not be melt inclusion rich support the occurrence of magma intrusion into cool regions of the lithosphere. Finally, turbulent flow during the movement of melts within the system, potentially during final ascent and eruption, may result in synneusis (7) and the formation of monomineralic glomerocrysts and attachment of plagioclase crystals. Eventual eruption (8) results in basalts that contain complex plagioclase crystal cargo, the textures of which can be related to both protracted growth histories and occurrence of specific processes within the plumbing system of the Gakkel Ridge.

## Electronic supplementary material

Below is the link to the electronic supplementary material.

Supplementary material 1 (XLSX 1274 kb)

Supplementary material 2 (PDF 8367 kb)

## Data Availability

All relevant data are included in the paper and the supplementary information.
