# Supplementary material for: The significance of plagioclase textures in mid-ocean ridge basalt (Gakkel Ridge, Arctic Ocean)
Source: Contrib Mineral Petrol. 2019 May 21;174(6):49. doi: 10.1007/s00410-019-1587-1 (PMC6530810; doi:10.1007/s00410-019-1587-1)
Supplement: Supplementary file 2 — Supplementary material 2 (PDF 8367 kb) [file 410_2019_1587_MOESM2_ESM.pdf]

The significance of plagioclase textures in mid-ocean ridge basalt (Gakkel Ridge, Arctic Ocean)

Emma N. Bennett[a]*, C. Johan Lissenberg[a] and Katharine V. Cashman[b]

[a] School of Earth and Ocean Sciences, Cardiff University, Park Place, Cardiff CF10 3AT, UK

[b] School of Earth Sciences, University of Bristol, Wills Memorial Building, Bristol BS8 1RJ, UK

Corresponding author email: bennette7@cardiff.ac.uk

Supplementary information containing: supplementary tables S1to S10; supplementary figures S1to S5; and information relating to rhyolite-MELTS modelling.

1. Supplementary tables

Table S1: Primary standards used for calibration

**Primary standards**	
**Element**	Standard
**Si**	Jadeite
**Al**	Jadeite
**Mg**	Diopside
**Ca**	Diopside
**Na**	Jadeite
**Fe**	Chromite
**K**	Orthoclase

Table S2: Individual plagioclase phenocryst and glomerocryst classification

Crystal type		Parameter		Scale	Category				
<---Mono-mineralic glomerocryst-->	Individual plagioclase phenocrysts	Individual/ component habit		-	Tabular	Acicular	Skeletal	Resorbed	
		Crystal form		-	Anhedral	Sub-anhedral	Subhedral	Sub-euhedral	Euhedral
		Zoning		-	Oscillatory	Patchy* (1-5 scale)	Reverse	Normal	Sector
		Resorption Melt inclusion habit	Stages	1-n	-	-	-	-	-
			Extent	1-5	-	-	-	-	-
			Location	-	Internal	External	External and internal	-	-
		Melt inclusion habit		-	Circular	Negative crystal	Elongate	Boxy	Amoeboid
		Surface attachment		-	Plagioclase	Olivine	Clinopyroxene	Spinel	-
		Included components		-	Point	Olivine	Clinopyroxene	Spinel	-
	Poly-mineralic glomerocrysts	Component contacts		-	Point	Planar	Embedded		
		Component configuration		-	Open	Closed	-	-	-
		Process		-	Late stage	Rip-up	-	-	-
		Mineral components		-	Plagioclase	Olivine	Clinopyroxene	Spinel	
		Zoning	Plagioclase/olivine/clinopyroxene	-	Oscillatory	Patchy	Normal	Reverse	Sector
		Habit	Plagioclase	-	Tabular	Skeletal	Acicular	Anhedral	
			Olivine/clinopyroxene	-	Euhedral	Skeletal	Subhedral	Anhedral	
			Spinel	-	Skeletal	Hopper	Vermiform	Chain	Euhedral

*** Patchy zoning is classified on a scale of 1-5**

Table S3 Individual plagioclase habit abundance, size and composition

Crystal habit	Individual crystals							Glomerocryst components	
	Total (%)	Size range (mm)	Average size (mm)	♦An range	♠Median maximum An	Zoning complexity		Mono-mineralic	Poly-mineralic
						Range	Median		
**Tabular**	61	0.07-14	2.2	42-87	79	0-8	2	51.8*	27*
**Resorbed**	30	0.15-13	2.5	52-86	78	0-10	2	16.9*	3*
**Skeletal**	7	0.21-7.68	1.6	50-83	68	0-5	1	0.6*	11*
**Acicular**	2	0.18-6.36	1.1	62-78	75	0-3	0	0*	0*
**Multi-habit**	-	-	-	-	-	-	-	30.7	59

♠ The maximum anorthite content is calculated for each crystal; the median of these values is then found for each crystal habit.

♦The minimum and maximum anorthite content of each crystal is determined. For each crystal habit the range of these values is then determined. This ncludes plagioclase cores, mottled cores, skeletal core, mantle, rims, and melt inclusion rims.

*Percentage of glomerocrysts composed of entirely one type of crystal habit

Table S4 Individual and glomerocryst zoning and zoning complexity

Zoning	Individual crystals (%)	Mono-mineralic glomerocrysts (%)	Poly-mineralic glomerocrysts (%)
**Unzoned**	8.1	4	3
**One type of zoning***	18.8	26	44
**Complex zoning♦**	73	70	53
**Zoning complexity (range)**	0-10	0-6**	0-3**
**Zoning complexity (average)**	2	2**	2**

**Values account for zoning in the entire glomerocrysts not its individual components

♦Includes Patchy zoning

*Excludes patchy zoning

Table S5 Types of zoning present in individual plagioclase and glomerocrysts

Zoning	Individual plagioclase zoning (%)	Mono-mineralic (%)	Poly-mineralic (%)
**Unzoned**	8.1	4	3
**Normal only**	1.4	2	10
**Reverse only**	11.1	7	5
**Oscillatory only**	6.1	11	30
**Sector only**	0.2	1	0
**Patchy only**	16.3	5*	2**
**Complex**	56.7	57	51

*Patchy zoning is present in 38% of all mono-mineralic glomerocrysts
**Patchy zoning is present in 25% of poly-mineralic glomerocrysts

Table S6 Percentage of individual plagioclase that contain each of the different zoning types

Zoning	%
Normal	35
Reverse	62
Patchy	43
Oscillatory	30
Sector	1

Table S7 Resorption location in individual and euhedral plagioclase and mono-mineralic glomerocrysts

Type of resorption	Type of crystal		
	Individual plagioclase (%)	Euhedral plagioclase (%)	Mono-mineralic glomerocrysts (%)
**Internal**	41	41	35
**External**	29	11	29
**Internal and external**	14	4	17
**None**	16	44	18

Table S8 Number of resorption events in each individual plagioclase habit category

Crystal habit	Average number of resorption events per crystal (%)	>/= 1 resorption event (%)	>/= 2 resorption event (%)
**Tabular**	1.22	85	29
**Resorbed**	1.33	100	26
**Skeletal**	0.34	31	2
**Acicular**	0.12	8	4

Table S9 Melt inclusion average and maximum size

Melt inclusion habit	Average size range (um)	Maximum size (um)
**Circular**	8-37	446
**Negative crystal**	7-41	342
**Elongate**	17-141	1610
**Boxy**	22-229	3220
**Amoeboid**	47-383	4947

Table S10 Glomerocryst configuration and component contacts

	Configuration (%)		Component contacts (%)		
**Glomerocryst type**	Open	Closed	Point	Planar	Embedded
**Mono-mineralic**	10	90	28*	51*	75*
**Poly-mineralic**	51	49	47*	18*	98*

*Percentage of glomerocrysts that contain that type of contact, not the percentage with only that contact type

2. Supplementary figures

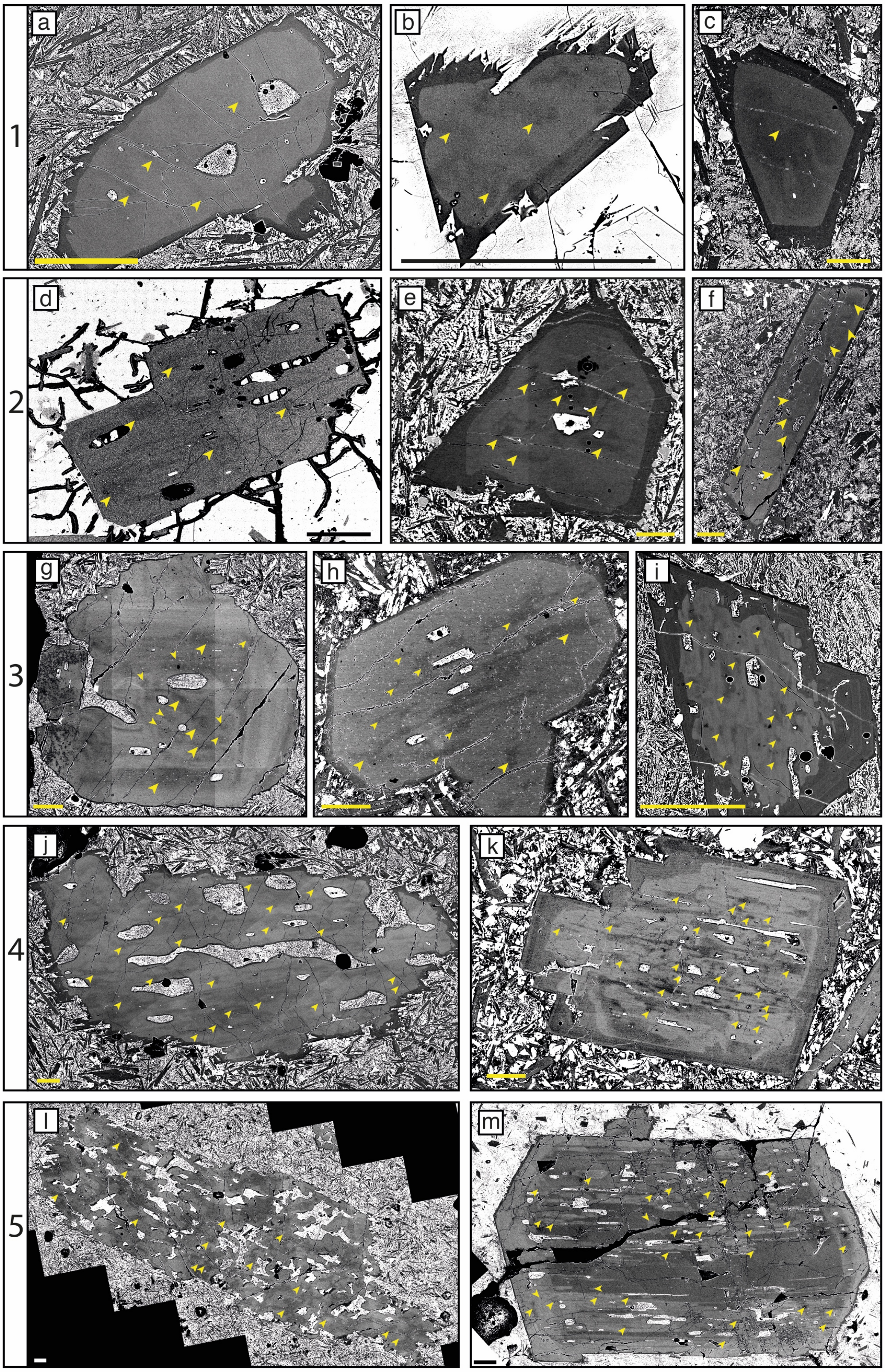

Fig. S1 Patchy zoning scale used to assign patchy zoning values to plagioclase crystal cargo. Note: patchy zoning is a continuous scale, hence multiple examples are shown for each category. Scale bars are all 2.5 mm

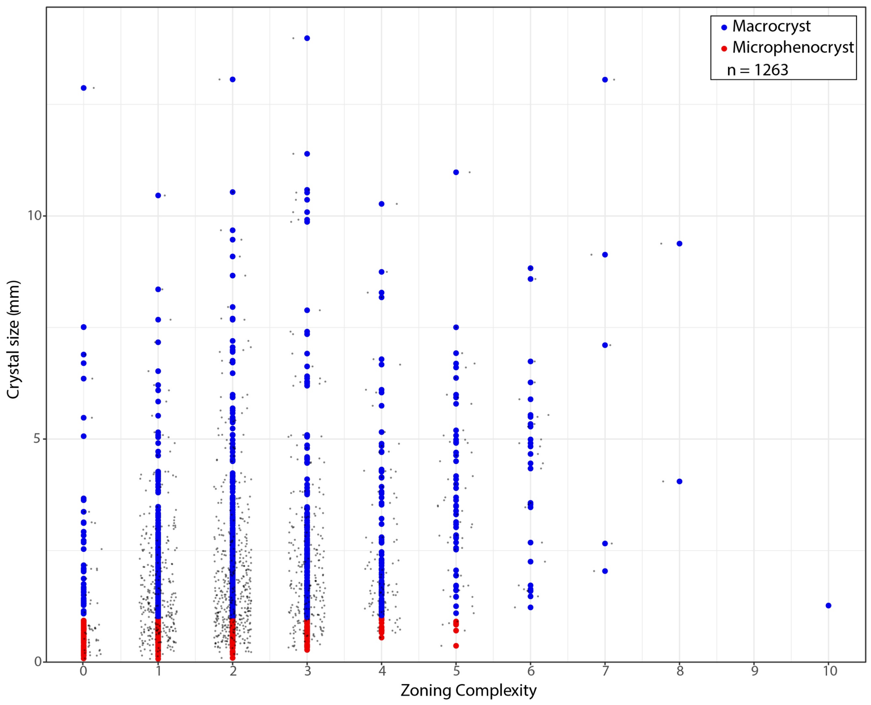

Fig. S2 There is no clear relationship between the size of individual plagioclase crystals size and zoning complexity. Small black points are the same as those in red and blue but have been plotted to show the distribution of the data.

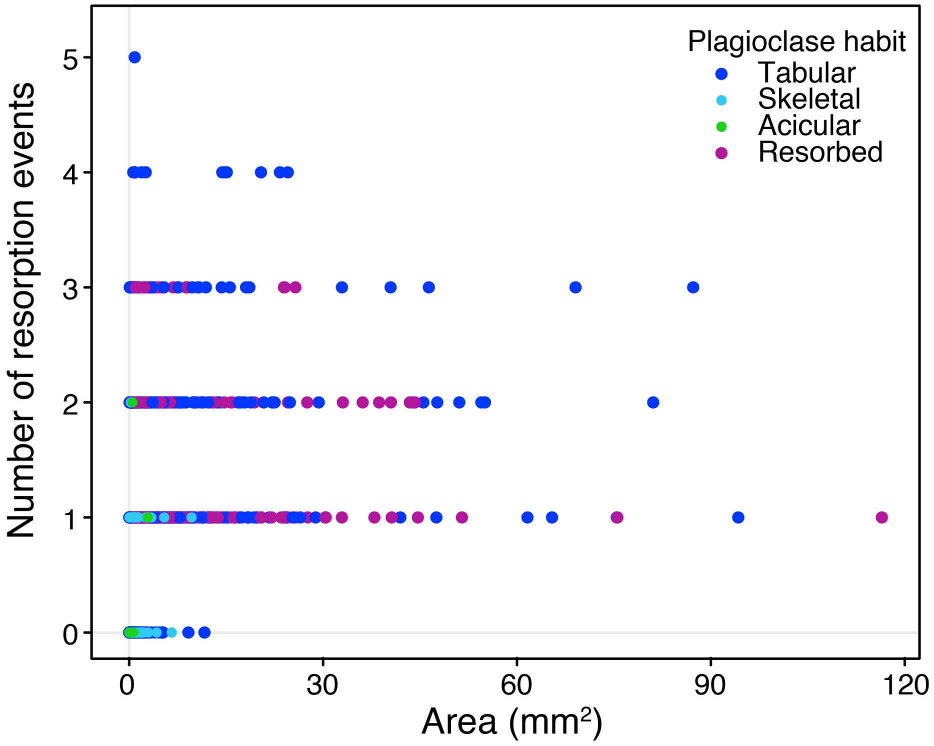

Fig. S3 Relationship between crystal area and number of resorption events.; there is no relationship between crystal area and number of resorption events. Note: because the crystal area was calculated using the maximum dimensions of the crystal and does not account for resorption, the crystal areas are maximums; the area of resorbed crsytals will be less than that calculated.

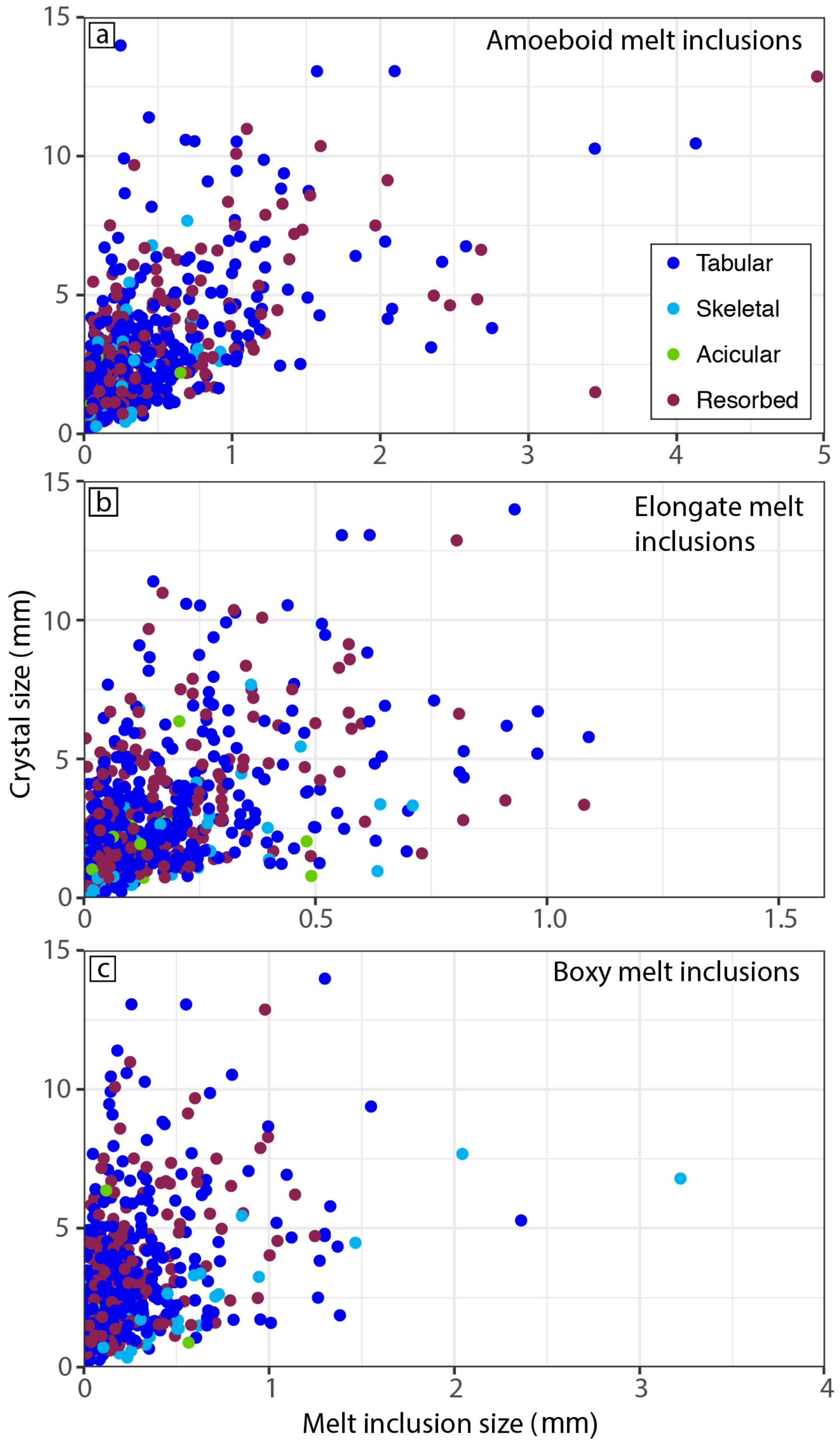

Fig. S4 Relationship between crystal size and size of amoeboid (**a**), elongate (**b**) and boxy (**c**) melt inclusions. For the three types of melt inclusions shown, there are weak positive correlations between crystal and melt inclusion size, larger crystals tending to have larger melt inclusions. **a** The largest amoeboid melt inclusions are situated in resorbed and tabular crystals. **b** Elongate melt inclusions are smaller than both amoeboid and boxy melt inclusions, with the largest elongate melt inclusions located in tabular and resorbed crystals. **c** Boxy melt inclusions have a similar size range to amoeboid inclusions, but the largest inclusion are located in skeletal and tabular crystals.

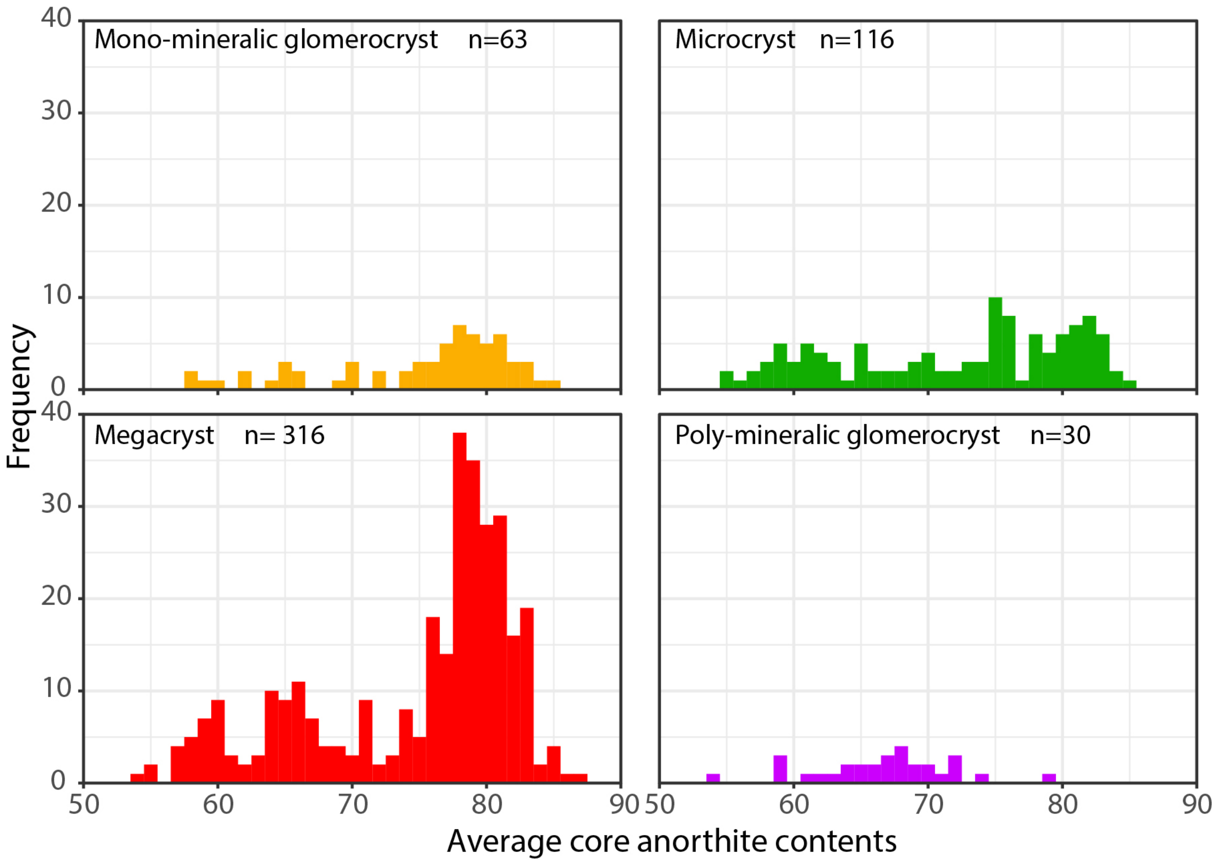

Fig. S5 Relationship between average core anorthite and crystal type. The range of average core anorthite for each crystal category overlap; the average core anorthite contents of poly-mineralic glomerocrysts is more restricted.

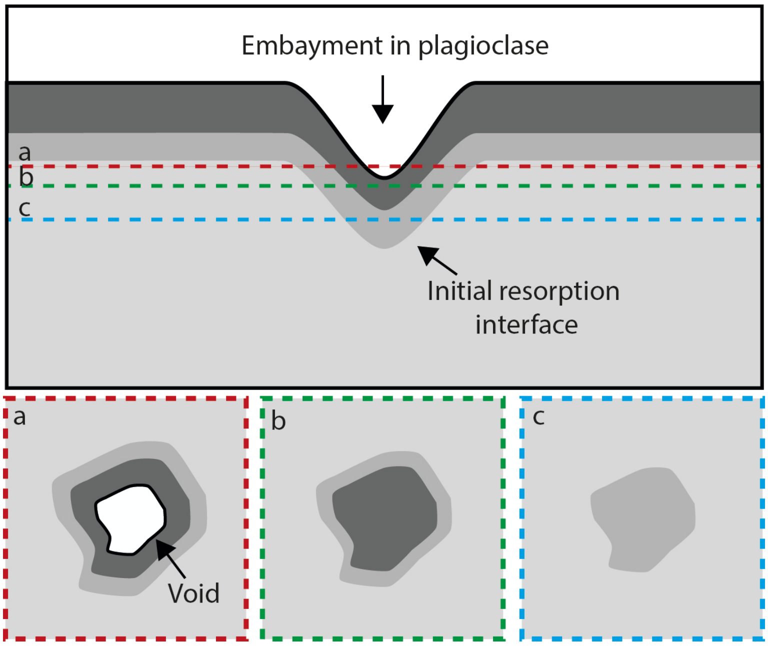

Fig. S6 Schematic showing the effect of sectioning on the observed plagioclase zoning pattern.

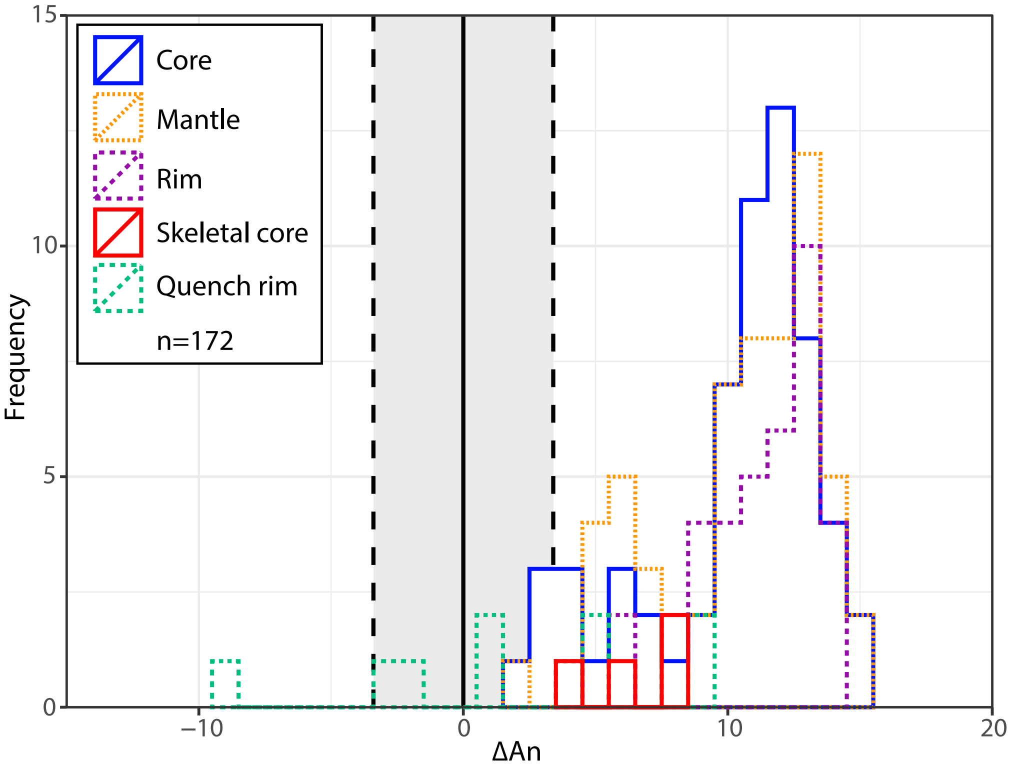

Fig. S7 Histogram of ΔAn demonstrating that plagioclase from dredge HLY0102-D27 show extensive disequilibrium regardless of the analysis location (e.g., core, rim etc.). The majority of analyses plot to the right in equilibrium with more primitive melt compositions than host glasses. Solid black vertical lines represent equilibrium (ΔAn=0); dashed lines represent ±5% (Grove et al. 1992) error on the average plagioclase anorthite content (An_{eq}) calculated using equations (1) and (2)

3. MELTS modelling

The pressure dependence of plagioclase anorthite content was determined using rhyolite-MELTS (Gualda et al. 2012). Six samples (HLY0102-050-043, POL0059-228-003, HLY0102-012-013, POL0059-306-026, HLY0102-095-037, HLY0102-091-004, HLY0102-028-HY, HLY0102-060-045, HLY0102-053-018), were run at 1-5 kbar and QFM-1, and the pressure dependence determined from the slopes of the pressure-An relationships; the average change in anorthite per kbar was 1.3, and was found to be essentially uniform between the different samples and across the pressure range investigated.

References

Gaulda GAR, Ghiorso MS, Lemons R V., Carley TL (2012) Rhyolite-MELTS: A modified calibration of MELTS optimized for silica-rich, fluid-bearing magmatic systems. J Petrol 53:875–890. doi: 10.1093/petrology/egr080
